# Embedded 3D Printing of Graphene Oxide‐Containing, Chemically Crosslinkable Poly(Ethylene Glycol) Inks

**DOI:** 10.1002/smsc.202500278

**Published:** 2025-11-08

**Authors:** Helena P. Ferreira, Monize C. Decarli, Duarte Moura, Rúben F. Pereira, Andreia T. Pereira, Lorenzo Moroni, Inês C. Gonçalves

**Affiliations:** ^1^ i3S – Instituto de Investigação e Inovação em Saúde Universidade do Porto Rua Alfredo Allen 208 4200‐135 Porto Portugal; ^2^ INEB – Instituto Nacional de Engenharia Biomédica Universidade do Porto Rua Alfredo Allen 208 4200‐135 Porto Portugal; ^3^ ICBAS – Instituto de Ciências Biomédicas Abel Salazar Universidade do Porto Rua Jorge de Viterbo Ferreira 228 4050‐313 Porto Portugal; ^4^ MERLN Institute for Technology‐Inspired Regenerative Medicine Department of Complex Tissue Regeneration Maastricht University Universiteitssingel 40 Maastricht 6229 ER The Netherlands; ^5^ Department of Biomaterials & Biomedical Technology University Medical Center Groningen/University of Groningen A Deusinglaan 1 Groningen AV 9713 The Netherlands

**Keywords:** additive manufacturing, anti‐adhesiveness, graphene oxide, poly(ethylene glycol) hydrogels, shape retention, support baths

## Abstract

The incorporation of graphene‐based materials into hydrogels enhances their mechanical, electroconductive, and antimicrobial properties, offering significant potential for biomedical applications. However, 3D printing graphene‐containing inks may present challenges because of their unsuitable shape retention or the fact that the concentration of the graphene component can hinder photocrosslinking. This study explores embedded 3D printing to process a chemically crosslinkable poly(ethylene glycol) ink with a high (4% w/v) graphene oxide concentration (PEG/GO). Given the PEG/GO ink's insufficient shape retention and slow crosslinking, various support baths are screened, with the microparticulate bath of the crystal self‐healing embedding bioprinting (CLADDING) method proving most effective. The interstitial solution of the CLADDING bath influences the mechanical properties of printed PEG/GO constructs. Multilayered PEG/GO cylindrical constructs with <500 μm filament width and up to 4.5 mm height (30 layers) are fabricated, presenting better tensile properties when printed within CLADDING in calcium chloride (vs. baths in crosslinking initiators). The surface of PEG/GO constructs is anti‐adhesive toward human foreskin fibroblasts, and their extracts are cytocompatible. Hence, embedded 3D printing emerges as an innovative strategy to surpass limitations of shaping graphene‐containing hydrogels into complex geometries, broadening the biomanufacturing possibilities for diverse biomedical applications requiring kPa‐range mechanical properties.

## Graphical abstract

Embedded 3D printing, particularly inside the crystal self‐healing embedding bioprinting support bath, allows for the printing of chemically crosslinkable poly(ethylene glycol) (PEG) hydrogels with high graphene oxide (GO) content into multilayered geometries with submillimeter resolution. PEG/GO hydrogels can be shaped into constructs with kPa mechanical properties, maintaining their anti‐adhesive surface and cytocompatibility (extracts' assay), prompting their use in biomedical applications.

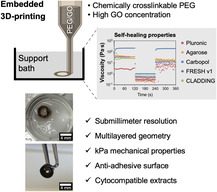

## 1 Introduction

Graphene is a single‐layer honeycomb lattice of sp^2^‐bonded carbon atoms with outstanding strength and electrical and thermal conductivity. Since its isolation in 2004 and being the subject of the 2010 Nobel Prize in Physics, graphene and its derivatives have conquered scientific and societal attention, securing a place among the most widely explored materials across a variety of fields.^[^ ^1^ ^]^ In the biomedical field, graphene‐containing biomaterials have been proposed for various applications, including drug delivery systems, phototherapies, and devices with load‐bearing, antimicrobial, or electroconductive functionalities for bone, neural, cardiovascular, cartilage, and musculoskeletal tissue engineering.^[^ ^2^, ^3^ ^]^ In particular, composites of polymers and hydrogels enriched with graphene oxide (GO, a form of graphene with oxygen‐containing groups on its surface) have received special attention, due to GO's higher hydrophilicity and overall greater biocompatibility than nonoxidized graphene forms.^[^ ^4^ ^]^ Graphene‐containing biomaterials may be processed through a variety of manufacturing techniques, from solvent casting, spray/dip/spin/perfusion coating, molding, electrospinning, extrusion‐based 3D printing, stereolithography, inkjet printing, selective laser sintering, or fused deposition modeling.^[^ ^5^ ^]^

Extrusion‐based 3D printing is a particularly useful additive manufacturing technique, since it allows the deposition of materials into complex geometries with great precision, from micrometer to millimeter resolution/dimensions, enabling to creation of tissue‐like structures, particularly using hydrogels.^[^ ^6^ ^]^ Recent advances in 3D printing of graphene‐containing biomaterials (including hydrogels) have been reviewed elsewhere.^[^ ^5^, ^6^, ^7^, ^8^ ^]^ Graphene and its derivatives have been used in printable inks for different purposes, ranging from tuning ink's rheological properties and improving the mechanical properties of hydrogels to imparting new biological features.^[^ ^6^ ^]^ Previous studies reported graphene‐containing inks with a wide range of concentrations (from as low as 0.025, up to 4% w/v GO) and different crosslinking mechanisms (physical vs. chemical, radical vs. light‐initiated) for a variety of applications.^[^ ^9^, ^10^ ^]^ Of notice, 3D printing with photocrosslinking can only be performed for inks with low GO concentrations (up to 0.8% w/v), since the ink becomes opaque with high GO content, preventing light penetration and activation of photoinitiators within the ink.^[^ ^11^ ^]^

However, 3D printing chemically crosslinkable biomaterials with higher GO contents could open new avenues for the shaping of materials with outstanding mechanical or electroconductive performance into products with complex geometries, for example.^[^ ^7^ ^]^ Such inks may pose significant challenges to 3D‐printing, since alternative crosslinking strategies (such as radical‐initiated mechanisms) have to be used. Additionally, although some authors have employed GO as a rheological modulator to increase the ink's viscosity, its final shape retention is still dependent on the polymer's viscosity and crosslinking time.^[^ ^12^, ^13^ ^]^ Indeed, many of these works deal with inks traditionally explored in 3D printing, such as alginate, which have, *per se*, acceptable/tunable shape retention, while inks with lower viscosities pose greater challenges.

In the past few years, embedded 3D printing has emerged as an alternative for processing inks with low viscosity and/or slow crosslinking kinetics. In this technique, the ink is extruded within a support bath that physically holds the printed filament until it is fully crosslinked. The support bath behaves as a solid component but flows when the applied stress surpasses its yield stress—i.e., when the printing needle is immersed. At this stage, the support bath fluidizes in the vicinity of the printing needle, which enables its free movement with subsequent extrusion and deposition of the ink in the designed pattern. After needle passage, the local stress decreases rapidly and the support bath recovers its initial solid‐like properties, self‐healing and maintaining the printed structures in place.^[^ ^14^ ^]^ Support baths already reported in the literature are mostly slurries of compacted microparticles, and include Pluronic F127 hydrogels, Carbopol gels, agarose fluid gels, gelatin‐based microparticle support baths—such as freeform reversible embedding of suspended hydrogels (FRESH v1 and v2) or crystal self‐healing embedding bioprinting (CLADDING)—, among others.^[^ ^14^, ^15^, ^16^, ^17^, ^18^, ^19^, ^20^, ^21^, ^22^, ^23^, ^24^, ^25^, ^26^, ^27^, ^28^, ^29^ ^]^

Therefore, embedded 3D printing may be a suitable technique for printing challenging GO‐containing inks with no or very low shape retention before crosslinking. However, only a few works have explored this alternative.^[^ ^15^, ^23^, ^30^ ^]^ Basu and colleagues showed that printing Pluronic F127/0.2% w/v GO ink in a Pluronic support bath allows for creation of unsupported, overhanging structures for millimeter‐thick filaments.^[^ ^15^ ^]^ In another work, Veerubhotla and Lee printed and photocrosslinked a pHEMA ink with 0.35% w/v methacrylated GO and PVP as a rheological modulator, producing cardiovascular stent tubes using a Carbopol support bath.^[^ ^23^ ^]^ Finally, Wang and colleagues showed how a GO dispersion in ethanol (6.2% w/w) could be printed within a Carbopol support bath and strengthened by chemical reduction, to form GO frameworks.^[^ ^30^ ^]^ Hence, the 3D printing of low viscosity, chemically crosslinkable inks with high GO concentrations is yet to be demonstrated, particularly for submillimeter resolutions.

The objective of this work was to explore the embedded 3D printing of an ink composed of poly(ethylene glycol) (PEG) with a high concentration (4% w/v) of GO, which could result in constructs with interesting mechanical properties for load‐bearing applications. PEG was chosen given its relevance for biomedical applications as a biocompatible, “blank slate” polymer, with tunable properties, for a plethora of applications.^[^ ^31^ ^]^ The PEG/GO ink can be crosslinked through a radical‐initiated mechanism using an ammonium persulfate (APS) and sodium metabisulfite (SMB) redox pair, which generates free radicals that activate the dimethacrylate extremity groups of PEG chains. This PEG/GO ink can generate strong, biocompatible, extremely hydrophilic, and anti‐adhesive 2D hydrogels, serving as a “blank slate” with tunable biophysicochemical properties.^[^ ^32^ ^]^ In particular, the incorporation of 4% w/v GO in PEG‐dimethacrylate hydrogels resulted in a 14‐fold increase in tensile strength, reaching up to 218 kPa.^[^ ^32^ ^]^ The PEG/GO ink was 3D‐printed to produce well‐defined, cylindrical constructs with millimeter‐diameter, submillimeter‐wall thickness, and variable length, by deposition of stacked concentric ring layers. This cylindrical geometry was chosen as a proof‐of‐concept because 1) it allows demonstration of submillimeter‐scale printing resolution; 2) it challenges the support bath by simultaneous two‐axis (*x* and *y*) movements to create the rings and successively disrupting it with the deposition of stacked rings; 3) it enables the creation of constructs simple enough to be handled, measured and mechanically tested. This study demonstrates the feasibility of printing PEG/GO inks with high GO concentrations by using a chemically crosslinkable strategy.

## 2 Results

### 2.1 PEG/GO Ink is Shear‐Thinning, but does not have Shape Retention when Printed on a Stage

To explore the printing process of a chemically crosslinkable ink with high GO content, a PEG/GO ink was prepared using 15% w/v PEG dimethacrylate (molecular weight 8 kDa) and 4% w/v GO. GO used in this ink was shown to be highly exfoliated (Figure S1A, Supporting Information), with a mean lateral size of 0.934 ± 0.408 μm (Figure S1B, Supporting Information) composed of two populations of particles (Figure S1C, Supporting Information). Elemental composition analysis revealed a high oxidation degree of 28.5% (Figure S1D, Supporting Information). GO zeta‐potential was −47.3 ± 0.51 mV, due to the presence of deprotonated oxygen‐containing groups at neutral pH; conductivity was very low, 0.0224 ± 0.0044 mS cm^−1^ (Figure S1E, Supporting Information), due to the disruption of graphitic sheet by the oxygen‐containing groups. The use of oxidized forms of graphene‐based materials (in this case, GO) is important to achieve good dispersion and homogeneity of an aqueous PEG‐based ink, as previously reported in the literature.^[^ ^4^ ^]^

GO serves as a nanofiller to mechanically reinforce hydrogels. Previous literature exploring the 3D printing of graphene‐containing inks focuses essentially on lower concentrations of GO, which result in inks that allow for the use of more advanced crosslinking mechanisms like photocrosslinking.^[^ ^11^ ^]^ In line with this, for GO‐containing PEG‐based inks, photocrosslinking appears to be possible only when GO concentration is lower than 1% w/v (Figure S2, Supporting Information). Hence, printing this PEG/4% w/v GO formulation (henceforth ‘PEG/GO’ ink) allows us to tackle the challenge of printing high GO content, chemically crosslinkable PEG‐based inks.

**Figure 1:**
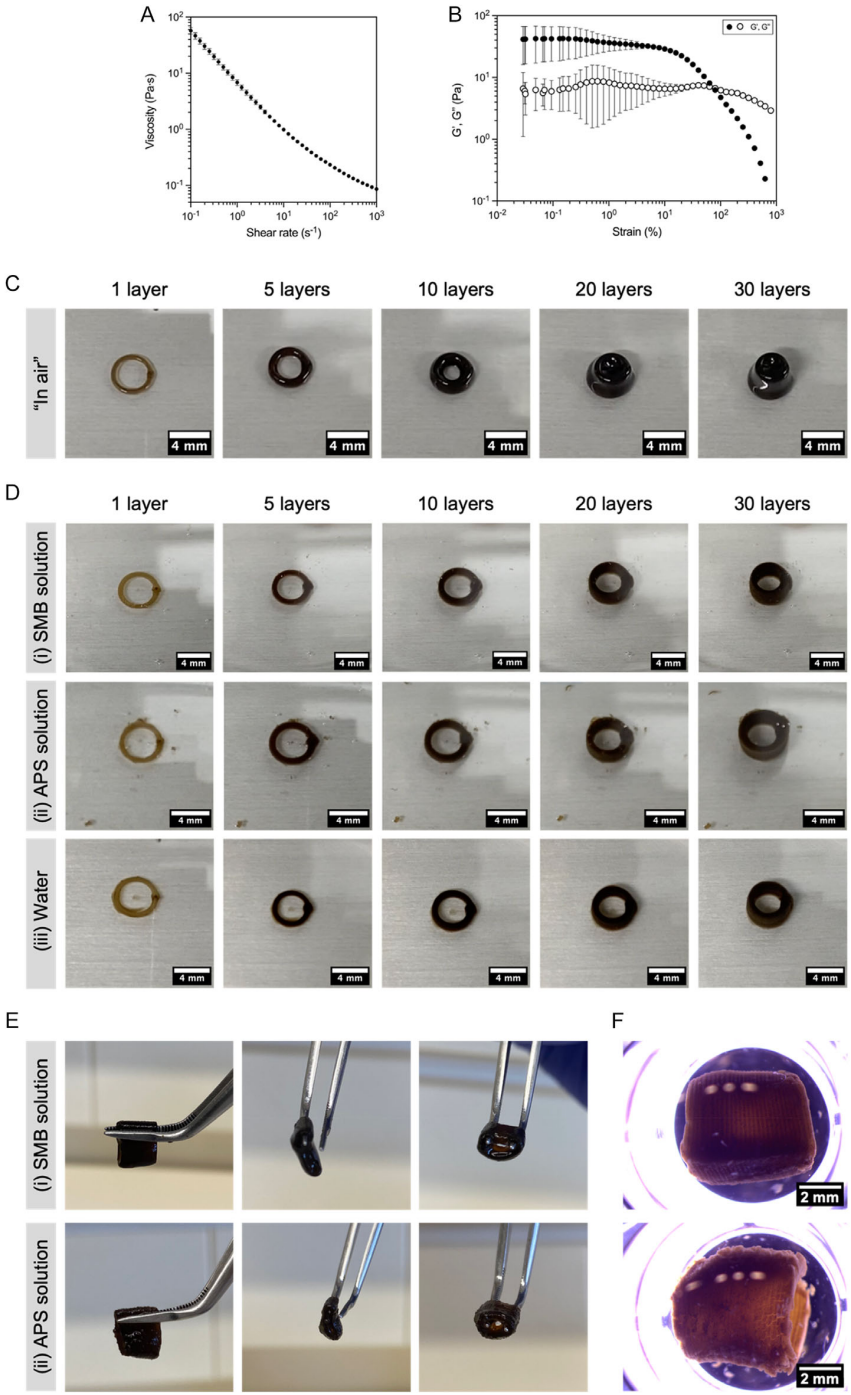
Rheological characterization of PEG/GO ink through A) flow sweep viscometry (10^−1^–10^3^ s^−1^ shear rate) and B) amplitude sweep oscillation test (10^−2^–10^3^% strain, 1 Hz constant oscillation frequency) (mean ± SD, *n* = 3 replicates). C) Extrusion printing of PEG/GO ink on printing stage (i.e., “in air”) in a cylindrical geometry (designed to have 4 mm inner diameter, 30 layers, printed with 27G needle, 0.15 mm interlayer spacing) with increasing number of layers (1, 5, 10, 20 and 30 layers). D) Extrusion printing of PEG/GO ink in a cylindrical geometry (4 mm inner diameter, 27G needle, 0.15 mm interlayer spacing) with increasing number of layers (1, 5, 10, 20 and 30 layers) within solutions of i) 1% w/v SMB, ii) 1% w/v APS or iii) water; scale bar: 4 mm. E) PEG/GO constructs after crosslinking and retrieval from i) SMB solution or ii) APS solution. F) Stereomicroscope images (side view) of PEG/GO constructs printed in SMB or APS solutions. Scale bar: 2 mm.

Rheological analysis showed the ink had a shear‐thinning behavior (**Figure** **1**A). The viscosity of our PEG/GO ink was around 50 Pa·s (at a low shear rate of 0.1 s^−1^, to simulate the period after extrusion and deposition), two orders of magnitude lower than that of 25% w/v Pluronic F127 (5000 Pa·s), a commonly explored ink with acceptable shape retention.^[^ ^15^ ^]^ This suggests that, while GO is a rheological modulator, the PEG/GO ink's viscosity might not be enough to allow for direct 3D‐printing on stage. Oscillatory tests showed that the PEG/GO ink presented a G′ higher than G″, even though the ink is not crosslinked (Figure 1B). This behavior has been reported for GO aqueous suspensions, and it is attributed to GO nanosheet self‐aggregation and formation of liquid‐crystalline phases for concentrations higher than a critical concentration (≈0.22 mg mL^−1^ or 0.022% w/v).^[^ ^33^, ^34^ ^]^ Yet, it is possible to see that the PEG/GO ink possesses a yield stress, suggesting these ordered structures disintegrate and the ink flows for high shear strains, such as those exerted during extrusion printing.

The PEG/GO ink was 3D‐printed through pneumatic extrusion to produce a cylindrical construct by deposition of stacked concentric ring layers on a standard printing platform. However, it was not possible to obtain a 30‐layer cylindrical construct (Figure 1C). The PEG/GO ink was capable of maintaining a circular shape when one layer was printed. However, as the number of layers increased, the ring started to collapse under its own weight (particularly visible for 10 layers). From 20 layers onwards, the construct lost its central aperture and was totally obstructed. The collapse of printed uncrosslinked structures happened in a matter of seconds, before the extrusion process was finished, as a result of poor shape retention.

One way to improve the viscosity of such inks could be to incorporate crosslinking initiators in the PEG/GO ink before extrusion. However, introducing the initiators in this traditional printing setup would be a challenge. The very high GO concentration prevents the use of knowingly fast photocrosslinking, and so gelation must be initiated using a redox pair, APS and SMB. Adding these redox initiators individually or together in the ink (each in a 0.1% w/v concentration) was not an option, since they both can initiate crosslinking,^[^ ^35^ ^]^ which would lead to needle clogging. In an attempt to create a larger printing time window while maintaining the PEG/GO ink fluid before crosslinking starts, we have tried decreasing the concentration of these initiators in the PEG/GO ink by half or a quarter (to 0.05% w/v and 0.025% w/v, respectively). Although this slightly increased the gelation time, the continuous crosslinking reactions still caused variations in the ink's viscosity in <30 min, making it impossible to define printing parameters that would remain constant and valid over a feasible amount of time (Figure S3, Supporting Information). Hence, the proposed PEG/GO formulation does not show suitable shape retention for printing lengthier geometries on a standard printing stage.

An additional hypothesis to surpass this limitation would be to print the constructs within solutions of dissolved initiators. This provides a way to include the redox initiators in the printing setup. PEG/GO constructs were printed in solutions of 1% w/v APS or SMB, as well as inside a water bath (after which 5 μL of highly concentrated APS and SMB solutions were pipetted on top of the construct, to fully ensure crosslinking) (Figure 1D). From those extrusion experiments, it was possible to conclude that a liquid media (even plain water) provided enough resistance to allow for shape retention of the PEG/GO filaments, and deposition of multiple layers without collapse. After printing, PEG/GO constructs printed in both SMB and APS solutions remained stable, while those printed in water rapidly disintegrated. This suggests that the presence of at least one redox initiator in the solution was enough to stabilize the constructs (either by decreasing spreading due to ionic strength or by initiating crosslinking alone). After crosslinking the constructs, they could be retrieved from the support bath. However, handling showed that these constructs were very weak and lacked structural robustness (Figure 1E). In closer observation, it was possible to see cracks in the constructs (Figure 1F). Of notice, printing in a solution with both APS and SMB was not pursued, because these redox initiators generate free radicals when they come in contact with each other, whose action has an unknown, but limited lifetime.^[^ ^36^ ^]^ Hence, to prevent losing radical‐generating action over time (before extrusion printing is finished, for example), this possibility was discarded.

To guarantee PEG/GO ink's shape retention and generate more robust constructs, embedded 3D printing using microparticulate support baths was explored.

### 2.2 Printing in a Support Bath Ensures PEG/GO Ink's Shape Retention, with CLADDING Yielding the Smoothest and Most Stable Filaments

**Figure 2:**
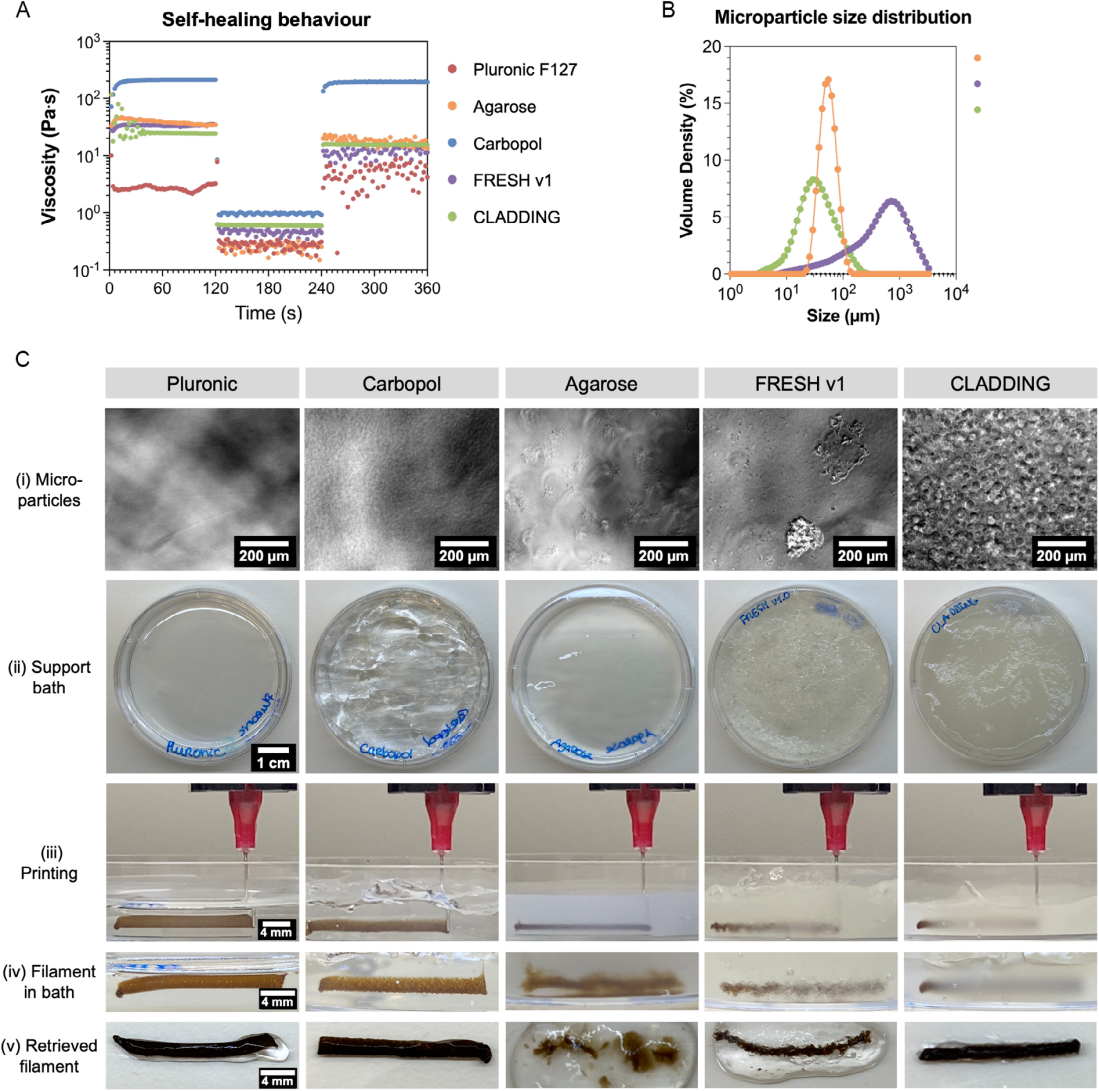
A) Rheological characterization of support baths through three‐step viscometry assay (2 min at 0.1 s^−1^ shear rate, 2 min at 100 s^−1^, and 2 min at 0.1 s^−1^). B) Microparticle size distribution of support baths, as volume density, for agarose, FRESH v1, and CLADDING support baths. Size distributions were not attainable for Pluronic and Carbopol support baths for technical reasons, since obscuration target values were never achieved. C) Comparative assessment of support baths reported in the literature. i) Phase‐contrast microscopy images of microparticles (1:80 dilution) from each support bath; scale bar: 200 μm. ii) Top view macroscopic images of produced support baths; scale bar: 1 cm. iii) Side view of 2‐cm PEG/GO filament printed within each support bath, immediately after extrusion and before needle retraction; scale bar: 4 mm. iv) Side view of 2‐cm PEG/GO filament printed in each support bath a few minutes after printing, before crosslinking; scale bar: 4 mm. v) PEG/GO filaments retrieved from each support bath, after crosslinking with APS and SMB; scale bar: 4 mm.

We started by selecting, producing, and characterizing some support baths reported in the literature, namely Pluronic F127, agarose fluid gel, Carbopol gel, FRESH v1, and CLADDING (**Figure** **2**). The self‐healing behavior of these hydrogels was evaluated by three‐step viscometry assays (Figure 2A). In these assays, the support bath is submitted to a sequence of low, high, and low shear stresses; the high shear stress stage simulates the passage of the needle during the printing process, while the following low shear stress stage showcases the closing of the groove caused by the needle. Our results suggest that Pluronic F127 structure was disrupted after being subjected to high shear stress, while Carbopol gels required a couple more seconds to recover the initial properties. The FRESH v1 and agarose baths almost recovered their initial properties, although revealing some instability in the third stage as well. Notably, the CLADDING support bath showed the better and quickest recovery of its initial properties after the high shear stress was applied.

The microparticles that compose these support baths were also characterized. From microparticle size distribution results (Figure 2B and Table S1, Supporting Information) evaluated by a DLS‐based technique, agarose support baths presented a microparticle population with little polydispersity, with 53.6 ± 0.1 μm Dv50 (median size). FRESH v1 presented larger microparticles within a wide range of sizes, from 78.4 ± 3.0 μm (at Dv10) and 1580 ± 102 μm (at Dv90). The CLADDING bath presented microparticle distribution with the lowest median size, of only 33.1 ± 0.8 μm, although slightly more polydisperse than agarose baths. Microparticle shape was visualized by phase‐contrast microscopic images (Figure 2Ci). It was not possible to visualize microparticles from Pluronic support baths, because this bath is composed of Pluronic F127 micelles with sizes reported between 20 and 80 nm.^[^ ^37^ ^]^ Microscopic images from Carbopol baths, resulting from the ionic gelation of Carbopol 940P micelles, suggest the presence of small microparticles. Agarose support baths, produced by the stirring of cooling agarose solutions, presented the irregularly shaped “hairy” particles reported before, with a fairly uniform size.^[^ ^26^ ^]^ FRESH v1, obtained from the mechanical blending of gelatin gels, was more heterogeneous, with particles ranging from extremely small microparticles to hundred‐micrometer‐wide, irregularly shaped particles with angular and sharp sides.^[^ ^28^ ^]^ The CLADDING support bath was composed of round‐shaped gelatin/gum arabic microspheres, and it is possible to visually distinguish larger microspheres and highly compacted smaller microspheres, corroborating the microparticle size distribution results.

After characterizing the produced support baths, their self‐healing behavior was evaluated by extruding PEG/GO short filaments (Figure 2Ciii). The PEG/GO filaments’ shape and integrity were assessed after extrusion (Figure 2Civ), as well as after crosslinking (ensured by pipetting 5 μL of highly concentrated APS and SMB solutions on top of the filaments) and retrieval (Figure 2Cv). As can be seen in the images (Figure 2Ciii), Pluronic and Carbopol support baths presented an imperfect groove closure after needle passage, as evidenced by the ink occupying the groove, in the vertical direction. These results are in accordance with the thixotropy assay results (Figure 2A). It has been reported that printing needles induce voids in Pluronic F127 support baths, which can only be filled by liquid Pluronic, while other works produce geometries with only little vertical overlay of printed filaments.^[^ ^15^, ^17^ ^]^ As for the Carbopol bath, although it was produced using Carbopol 974P—previously proposed for printing of pHEMA/methacrylated GO—it does not self‐heal properly, suggesting that Carbopol type influences not only filament smoothness and resolution, but also the rheological properties of the bath.^[^ ^20^, ^23^ ^]^ The agarose, FRESH v1, and CLADDING support baths presented very good self‐healing ability, as can be seen by the total closure of the groove created by the needle. Unexpectedly, the PEG/GO filament diffused greatly and sagged down the agarose bath, only a few minutes after extrusion, which led to incomplete filament crosslinking and subsequent disintegration. This could be due to the overly fluid character of the agarose bath, also evidenced in the high shear middle‐stage of the thixotropy assay. FRESH v1 yielded irregular filaments, difficult to retrieve without disintegrating, due to the high polydispersity of this bath's microparticles. Finally, the CLADDING support bath allowed to stably extrude and crosslink the PEG/GO filament, enabling easy retrieval. In summary, CLADDING works as the best support bath to print PEG/GO given its superior self‐healing behavior (contrary to baths like Pluronic or Carbopol, composed of orderly structures of nano/microparticles) and yielding the smoothest filaments, due to their spherical microparticles with lower diameters (contrary to baths like agarose or FRESH v1, which have irregular or larger microparticles). Previous work published by our team defined a clear parametric window and specific class of gelatin to produce high yields of CLADDING support bath, with improved self‐healing properties and ability to support the printing of inks with low viscosity and no shape retention.^[^ ^29^ ^]^ Therefore, the CLADDING support bath was selected as the best support bath to print the PEG/GO ink.

**Figure 3:**
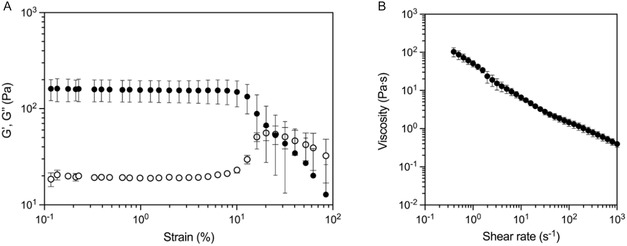
Rheological characterization of APS‐washed CLADDING support bath through A) amplitude sweep oscillation test (10^−1^–10^2^% strain, 1 Hz constant oscillation frequency) and B) flow sweep viscometry (10^−1^–10^3^ s^−1^ shear rate) (mean ± SD, *n* = 3 replicates).

The CLADDING support bath is composed of gelatin and gum arabic microparticles, stabilized at the surface by Pluronic; these microparticles are produced by a multistep coacervation process. After production, the CLADDING coacervate must be dispersed in a solution to form a microparticle colloid as the final support bath. For printing optimization, we selected a 1% w/v APS solution. Because printing PEG/GO ink in redox initiators’ liquid solutions appeared to generate less ink spreading (vs. distilled water) (Figure 1D), it was hypothesized that a similar effect would be verified for the solution where the CLADDING support bath was dispersed. Hence, this strategy might help achieve a better printing resolution, either by decreasing ink spreading or accelerating crosslinking. We set to characterize in detail the CLADDING in APS support bath produced in‐house. The CLADDING coacervate was dispersed in the APS solution, centrifuged, and compacted to obtain a colloid microparticle support bath. This CLADDING in APS bath was rheologically characterized. Amplitude sweep tests (**Figure** **3**A) have shown that, at low deformation strains, the bath had a solid‐like behavior. At higher deformation strains, the bath exhibited a yield‐stress behavior, where the viscous component surpasses the elastic component, showing the bath could locally fluidize when the needle penetrates it, and the local stress is higher than the yield stress. This yield stress has been calculated at 1.24 Pa. Additionally, the bath had a shear‐thinning behavior, as expected (Figure 3B). Therefore, the CLADDING in APS support bath was shown to be adequate for the embedded 3D printing of PEG/GO ink.

### 2.3 CLADDING Support Bath Allows for the Precise Control of PEG/GO Filament Width and Fabrication of Multilayered Structures

**Figure 4:**
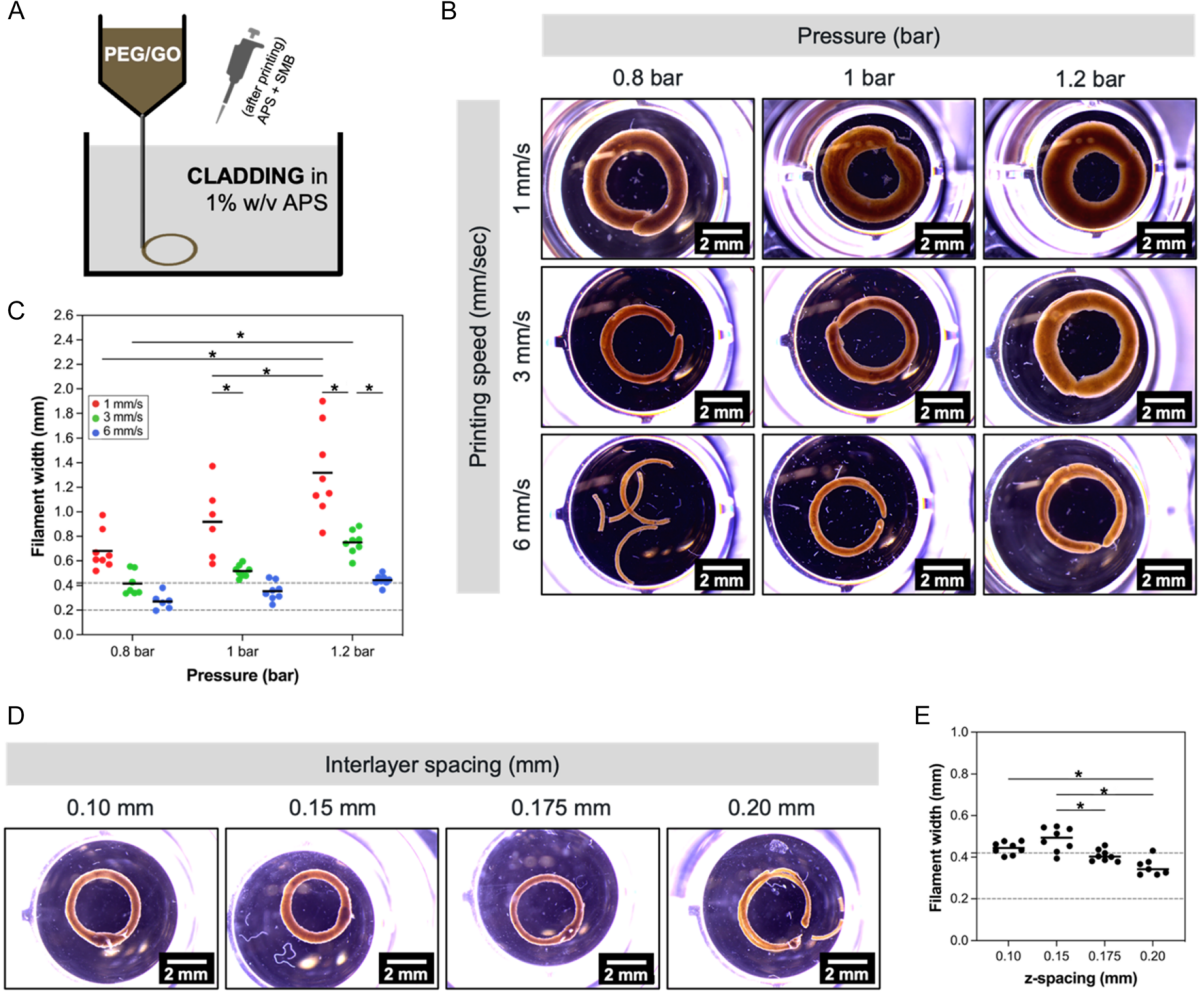
A) Schematic printing setup: PEG/GO ink (without any initiator) was pneumatically extruded into the CLADDING support bath, and crosslinking initiators (APS and SMB) were pipetted post‐extrusion, after which crosslinking was allowed to proceed overnight. B) Stereomicroscope images of PEG/GO 3‐layer rings printed with different extrusion pressures and printing speeds (interlayer spacing: 0.2 mm). Scale bar: 2 mm. C) Filament width of PEG/GO 3‐layer rings printed with different extrusion pressures and printing speeds (measured from stereomicroscope images). Dotted lines correspond to 27G needle inner (0.2 mm) and outer (0.42 mm) diameters. Mean values, *n* > 6 replicates, Two‐way ANOVA, Tukey's multiple comparisons test, *p < 0.05. D) Stereomicroscope images of PEG/GO 3‐layer rings printed with different interlayer spacing (extrusion pressure: 1 bar, printing speed: 6 mm s^−1^). Scale bar: 2 mm. E) Filament width of PEG/GO 3‐layer rings printed with different interlayer spacing (measured from stereomicroscope images). Dotted lines correspond to 27G needle inner (0.2 mm) and outer (0.42 mm) diameters. Mean values, *n* = 8 replicates, One‐way ANOVA, Tukey's multiple comparisons test, *p < 0.05.

For the optimization of the setup and procedure for 3D printing of PEG/GO ink, the CLADDING in APS support bath was used. The PEG/GO ink was pneumatically extruded within this support bath in a layer‐by‐layer fashion to produced cylindrical constructs with 4 mm inner diameter, 0.5 mm wall thickness and variable length; after extrusion, 5 μL of 20% w/v APS and SMB initiator solutions were pipetted on top of the construct, and these were left overnight to ensure crosslinking was completed (**Figure** **4**A).

To achieve a cylindrical construct with 0.5 mm wall thickness, printing was performed with a blunt‐end 27G needle, and the filament width was defined by the needle's diameter, extrusion pressure, and printing speed. Therefore, it was essential to optimize these parameters. Extrusion pressure ranged from 0.8 to 1.2 bar, while printing speed was tested between 1 and 6 mm s^−1^. Lower extrusion pressure, as well as higher printing speed, resulted in thinner filaments, as can be seen by stereomicroscope images of 3‐layer rings and the corresponding measured filament widths (Figure 4B). Of notice, for thicker filaments printed slowly (at 1 mm s^−1^), a higher variation of the filament width was observed for all tested pressures; additionally, the thicker the filaments, the larger the standard deviations (filaments are 0.68 ± 1.55 mm thick for 0.8 bar, 1 mm s^−1^, while being 1.32 ± 0.366 mm thick for 1.2 bar, 1 mm s^−1^). For such low printing speeds, there was a considerable ink accumulation, which also probably led to greater local disturbances in the support bath printing vicinity and might explain the higher variance observed. When comparing the filaments width and the printing needle's own inner and outer diameters (0.2 and 0.42 mm, respectively), it was possible to see filaments thinner than 0.42 mm (i.e., the needle's outer diameter) were not stable, and prone to delamination (for example, 0.27 ± 0.07 mm filaments printed at 0.8 bar, 6 mm s^−1^). The smallest filaments obtained while maintaining stability were 0.42 ± 0.09 mm thick (obtained for 0.8 bar, 3 mm s^−1^). This could be explained by the 0.2 mm interlayer spacing (initially arbitrarily set to print these rings), which was close to the filament width.

The next parameter to be optimized was interlayer spacing, which ranged between 0.1 and 0.2 mm (Figure 4C). Decreasing the interlayer spacing resulted in small but statistically significant differences in the filament width. The greatest differences were noted when decreasing from 0.2 to 0.15 mm, while the tendency was lost between 0.15 and 0.10 mm. From these results, an interlayer spacing of 0.15 mm was selected over 0.10 mm (to decrease the printing time when moving into lengthier constructs). In conclusion, these results show that the CLADDING bath allows for precise control of filament width.

**Figure 5:**
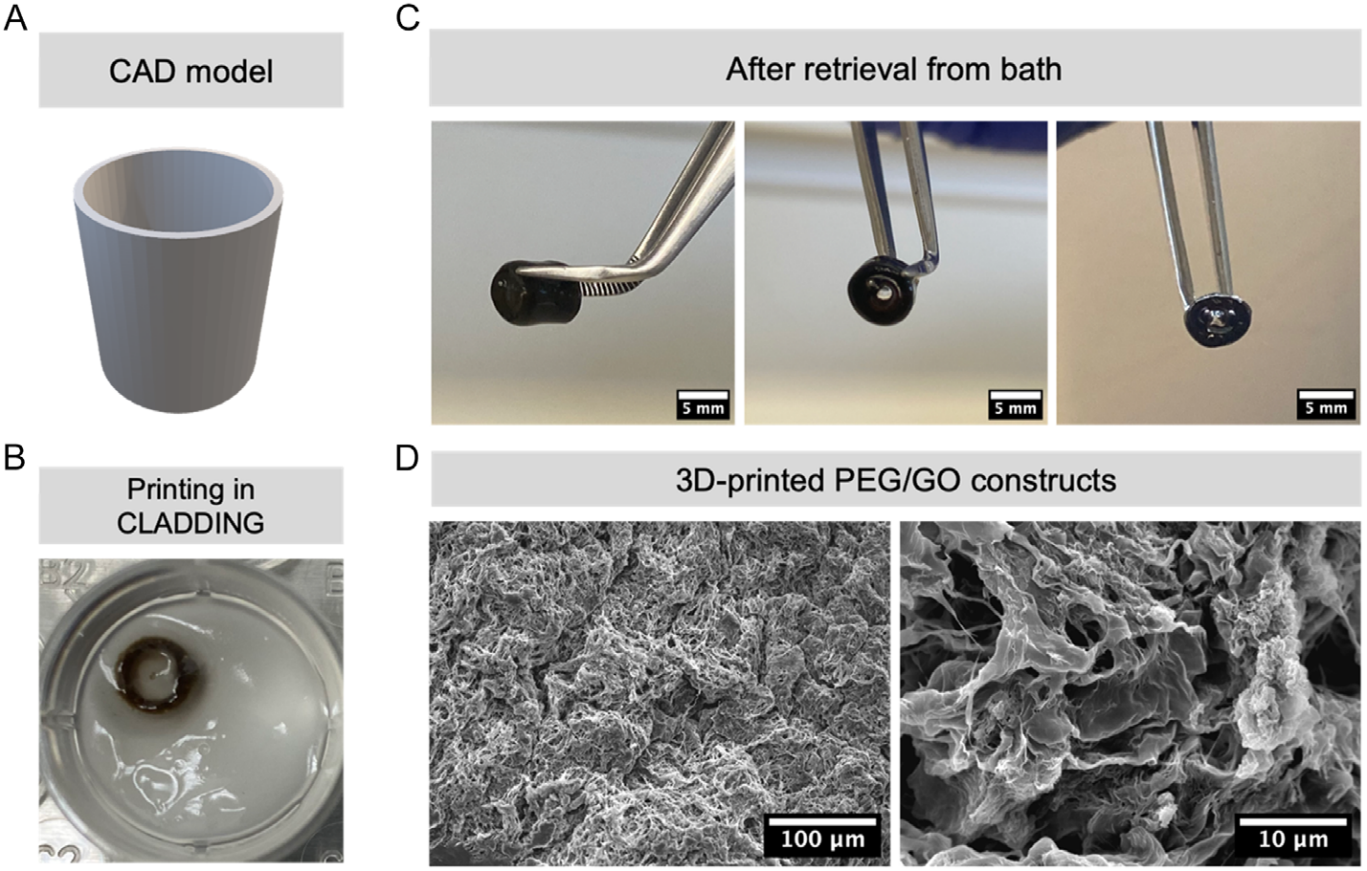
A) CAD model of a cylindrical construct with 4 mm internal diameter and 5 mm length. B) Top view of PEG/GO cylindrical construct printed inside CLADDING support bath, immediately after extrusion (extrusion pressure: 1 bar, printing speed: 6 mm s^−1^, z‐spacing: 0.15 mm, 30 layers). C) PEG/GO cylindrical constructs after crosslinking and retrieval from CLADDING support bath. Scale bar: 5 mm. D) SEM images of the inner surface of 3D‐printed PEG/GO constructs after retrieval from the support bath.

After optimizing these parameters, it was possible to print high aspect‐ratio structures, particularly cylinders with 4 mm inner diameter, 0.5 mm wall thickness, and 4.5 mm length (30 layers) (**Figure** **5**A). A notable retention of the shape of the PEG/GO cylinder when printed in the CLADDING in APS could be seen (Figure 5B), contrary to what happened when trying to print on a printing stage (i.e., “in air”, Figure 1C). It was possible to crosslink the PEG/GO ink within the support bath, release the PEG/GO construct from the bath without damage, wash and handle it with tweezers (Figure 5C). Scanning electron microscopy analysis of these constructs reveals a porous surface and irregular morphology (Figure 5D), contrary to bulky 2D PEG/GO hydrogels produced by molding (Figure S4, Supporting Information). While some roughness on the 3D‐printed constructs may also be caused by GO sheets, these have a more intricate morphology caused by the 3D printing process inside the CLADDING support bath. Such pores and irregularities in the structure are likely caused by interactions between the PEG/GO hydrogel and the CLADDING support bath, either in its microparticle form or by entanglement of polymeric chains of bath residues with the PEG/GO ink, during or after printing and crosslinking.^[^ ^32^ ^]^ Additionally, no particular orderly structures can be observed on the 3D‐printed PEG/GO constructs, with the formed hydrogel and the GO sheets contained in it having a rather random disposition. This is also in line with the observations on PEG/GO ink rheology: for higher shear stresses, as those exerted during pneumatic extrusion, the flow of the PEG/GO ink causes the possible liquid‐crystalline phases to disrupt, leading to a random disposition of GO sheets in the final printed hydrogel (Figure 1B). Additionally, these constructs were stable, as evaluated by their morphology and structural integrity up to at least 30 days postprinting (Figure S5, Supporting Information).

### 2.4 CLADDING Support Bath Solution Influences PEG/GO Constructs’ Mechanical Properties

**Figure 6:**
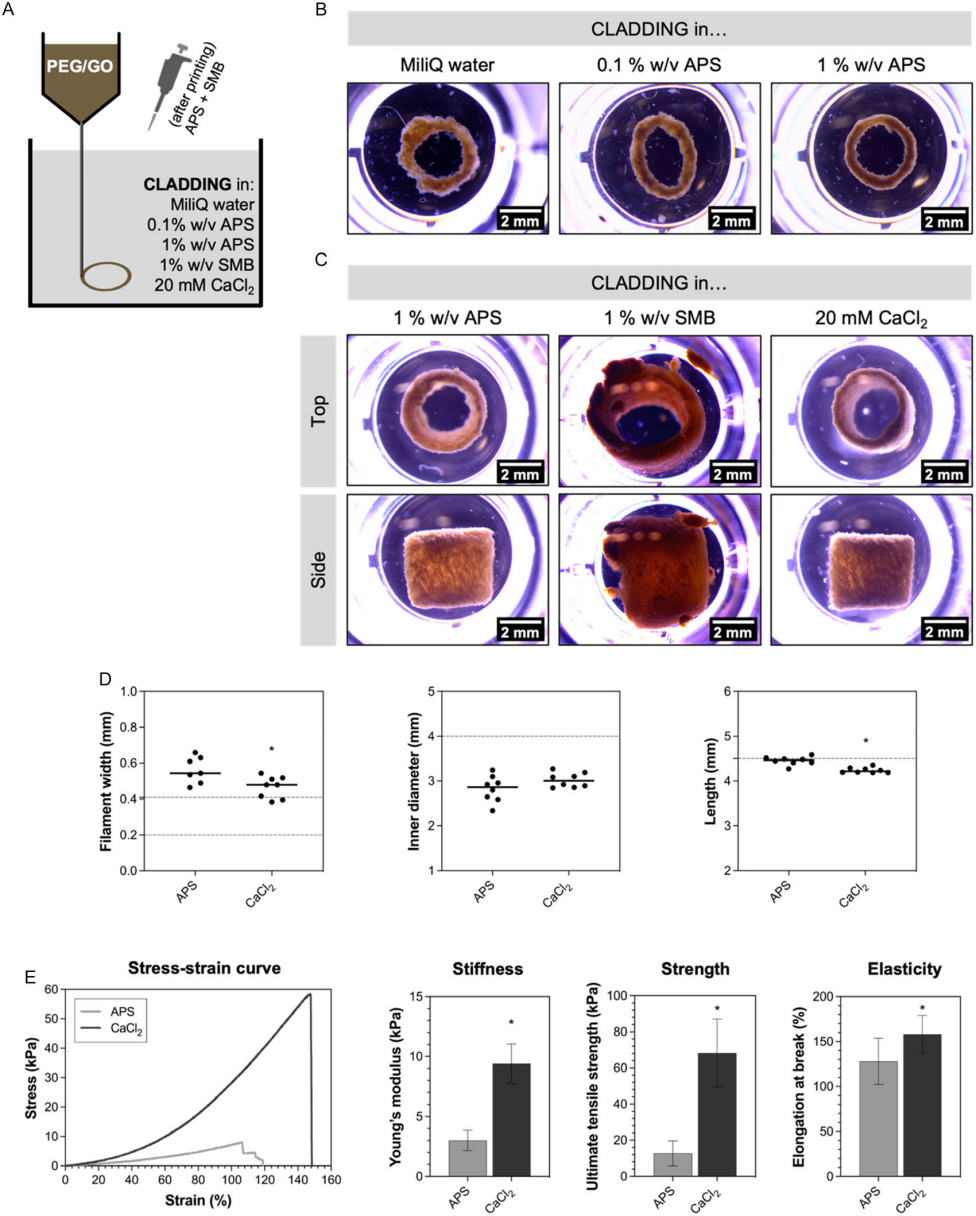
A) Schematic printing setup: PEG/GO ink (without any initiator) was pneumatically extruded into CLADDING support baths prepared in either MiliQ water, 0.1% w/v APS, 1% w/v APS, 1% w/v SMB or 20 mM CaCl_2_; crosslinking initiators (APS and SMB) were pipetted post‐extrusion, after which crosslinking was allowed to proceed overnight. B) Stereomicroscope images of PEG/GO 3‐layer rings printed in CLADDING baths in MiliQ water, 0.1% w/v APS or 1% w/v APS (extrusion pressure: 1 bar, printing speed: 6 mm s^−1^, interlayer spacing: 0.15 mm); scale bar: 2 mm. C) Stereomicroscope images (top and side views) of PEG/GO constructs printed in CLADDING baths in 1% w/v APS, 1% w/v SMB or 20 mM CaCl_2_ (extrusion pressure: 1 bar, printing speed: 6 mm s^−1^, z‐spacing: 0.15 mm, 30 layers); scale bar: 2 mm. D) Filament width and inner diameter (measured from stereomicroscope images top view images) and length (measured from stereomicroscope images side view images) of PEG/GO constructs printed in CLADDING baths in APS or CaCl_2_. In filament width graph, dotted lines correspond to 27G needle inner (0.2 mm) and outer (0.42 mm) diameters. In inner diameter and length graphs, dotted lines correspond to target dimensions (4 mm inner diameter and 4.5 mm length, respectively). Mean values, *n* = 8 replicates, One‐way ANOVA, *p < 0.05. E) Representative stress–strain curve of PEG/GO constructs submitted to a tensile test until rupture; Young's modulus, UTS and elongation at break of PEG/GO constructs printed in CLADDING baths in APS or CaCl_2_; mean ± SD, *n* = 8 replicates for each condition, parametric *t*‐test, *p < 0.05.

After optimization of 3D printing parameters to achieve the target geometry and dimensions, the influence of the support bath interstitial solution on printing resolution and constructs’ final properties was explored. We started by dispersing the CLADDING coacervate in different solutions and producing support baths in either MiliQ water, 0.1%, and 1% w/v APS, for subsequent printing of PEG/GO 3‐layer rings. Our results show that PEG/GO filaments printed in baths with MiliQ were highly irregular, while those printed in baths with APS were smoother. Importantly, the filament smoothness increased with increasing APS concentration (**Figure** **6**B). We also investigated the preparation of CLADDING support baths with higher APS concentrations (3% and 10% w/v), but it led to a great disruption of the support bath and liquification, rendering it unsustainable for printing. Next, we evaluated PEG/GO constructs 3D‐printed within CLADDING in either one of the redox initiators (APS or SMB) or a CaCl_2_ ionic solution (Figure 6C). Although the CLADDING bath in SMB allowed for the printing and crosslinking of PEG/GO constructs, the constructs were swelled, extremely fragile after retrieval from the support bath, and were disrupted by simple handling. Therefore, this option was not pursued further. On the other hand, PEG/GO constructs printed in CLADDING baths in APS or CaCl_2_ were robust and could be easily handled. Of notice, pipetting the 20% w/v APS and SMB initiators on top of the constructs post‐extrusion was enough to allow for gelation across the entire length of the constructs, either because these small‐molecule solutions can diffuse through the solution of the support bath or because the generated methacrylate free radicals can propagate longitudinally throughout the entire construct.^[^ ^38^ ^]^ When measuring the dimensions of these constructs (Figure 6D and Table S2, Supporting Information), it was possible to observe that 30‐layer constructs from CLADDING in APS were only slightly thicker (0.56 ± 0.07 mm) than 3 layer‐rings (and 0.48 ± 0.06 mm), which could indicate a slight weight‐induced coalescence effect. Side‐view stereomicroscope images of the constructs (Figure 6C) show that constructs from CLADDING in CaCl_2_ appeared more densely packed than constructs from CLADDING in APS. Comparing the obtained constructs for both support baths with the projected dimensions, it is possible to observe that constructs printed in CLADDING in CaCl_2_ had slightly lower wall thickness and length, while having similar inner diameter to those printed in CLADDING in APS. Finally, we set to evaluate the mechanical properties of these constructs, performing tensile tests until rupture. Stress–strain curves show that 3D‐printed PEG/GO constructs had an almost exclusively elastic behavior, with no plastic deformation before rupture (Figure 6E). Focusing on the stress‐strain curve of the samples from CLADDING in CaCl_2_ baths, the slope of the tangent to the curve increased with strain, which is indicative of compression of hydrogel network as the steel pins pull apart. PEG/GO constructs printed in CLADDING in CaCl_2_ were stiffer than those printed in CLADDING in APS (9.4 ± 1.6 vs. 3.0 ± 0.9 kPa, respectively), as well as stronger (68.2 ± 18.8 vs. 12.7 ± 6.9 kPa, respectively) and sustained higher elongations before tearing (158.0 ± 21.0 vs. 128.0 ± 25.7 kPa, respectively) (Figure 6E). Considering the cylindrical geometry of the constructs, besides the aforementioned properties, it is possible to calculate properties such as burst pressure (i.e., pressure of an internal fluid that causes bursting of the conduit) or compliance (i.e., ability of a conduit to deform/expand according to the internal pressure) (Figure S6, Supporting Information). The burst pressure of constructs printing in CLADDING in APS was 15.36 ± 6.40 mmHg, while that of constructs printing in CLADDING in CaCl_2_ was 60.03 ± 11.74 mmHg. Compliance was 776.1 ± 205.5%/100 mmHg for constructs from CLADDING in APS, but only 394.9 ± 54.94%/100 mmHg for constructs from CLADDING in CaCl_2_. Because PEG/GO constructs printed in CLADDING in CaCl_2_ had the best mechanical properties, these were selected for further studies.

### 2.5 PEG/GO Constructs Produced by Embedded 3D Printing have an Antiadhesive Surface and are Cytocompatible

**Figure 7:**
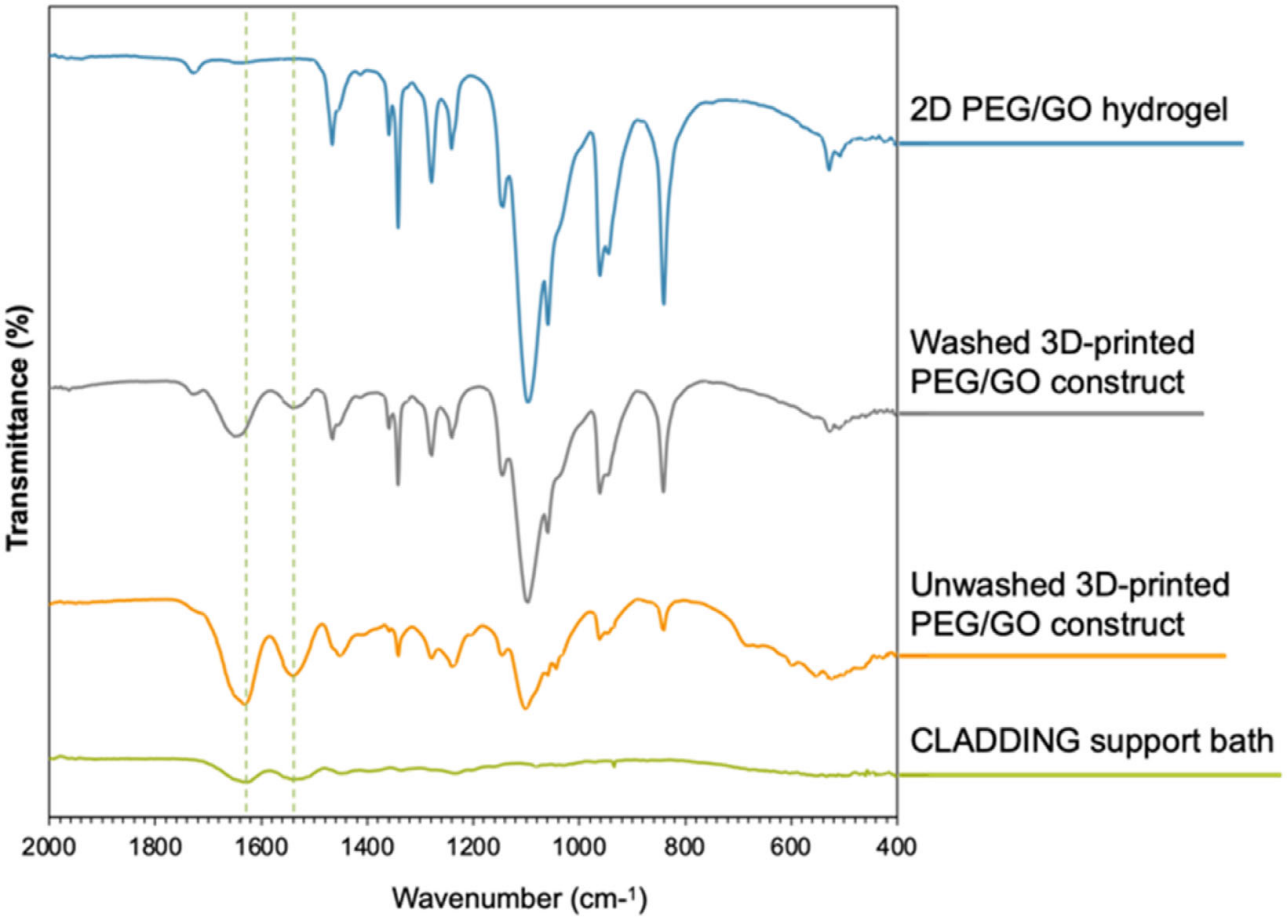
Representative FTIR‐ATR spectra of (top to bottom): 2D PEG/GO hydrogels (blue line), washed 3D‐printed construct (gray line), unwashed 3D‐printed construct (orange line), and CLADDING support bath (green line).

PEG/GO 2D hydrogels are highly antiadhesive (much like neat PEG hydrogels) against mammalian cells.^[^ ^32^ ^]^ In order to determine whether the embedded 3D printing process using the CLADDING support bath could affect the surface properties of the PEG/GO constructs, the chemical composition and anti‐adhesiveness of these constructs were evaluated. After removing the constructs from the CLADDING in CaCl_2_, they were washed overnight with distilled water at 37 °C to melt the gelatin, breaking apart the microparticles, and removing the surrounding support bath. After this washing process, we have visually verified that residual support bath can be found on the 3D‐printed constructs. To evaluate the extent of this washing process, the surface of 3D‐printed PEG/GO constructs before and after washing was assessed by Fourier‐transform infrared spectroscopy (FTIR)‐attenuated total reflection (ATR) spectroscopy and compared against spectra of PEG/GO hydrogels produced by molding (**Figure** **7**). We also attempted to acquire FTIR spectra for the CLADDING support bath, but, because this is a microparticulate colloid, the sample was very fragile and prone to disintegration when dried, which made spectrum acquisition difficult. Nonetheless, it was possible to observe two broad bands around 1530 and 1640 cm^−1^, though with little resolution. These bands can be attributed to amide bonds of gelatin.^[^ ^39^ ^]^ Analyzing the FTIR spectra of 3D‐printed PEG/GO constructs, it was possible to identify new peaks at 1533 and 1642 cm^−1^, which are aligned with the amide bonds’ peaks characteristic of the CLADDING support bath. These peaks cannot be found on the FTIR spectra of 2D PEG/GO hydrogels produced by molding, further confirming they are the result of the embedded 3D printing process. Additionally, these peaks are more intense in the unwashed 3D‐printed constructs, reinforcing the hypothesis that they are probably characteristic of support bath remnants. Of notice, although the washing process contributes to a decrease in their intensity, it does not completely eliminate support bath remnants.

**Figure 8:**
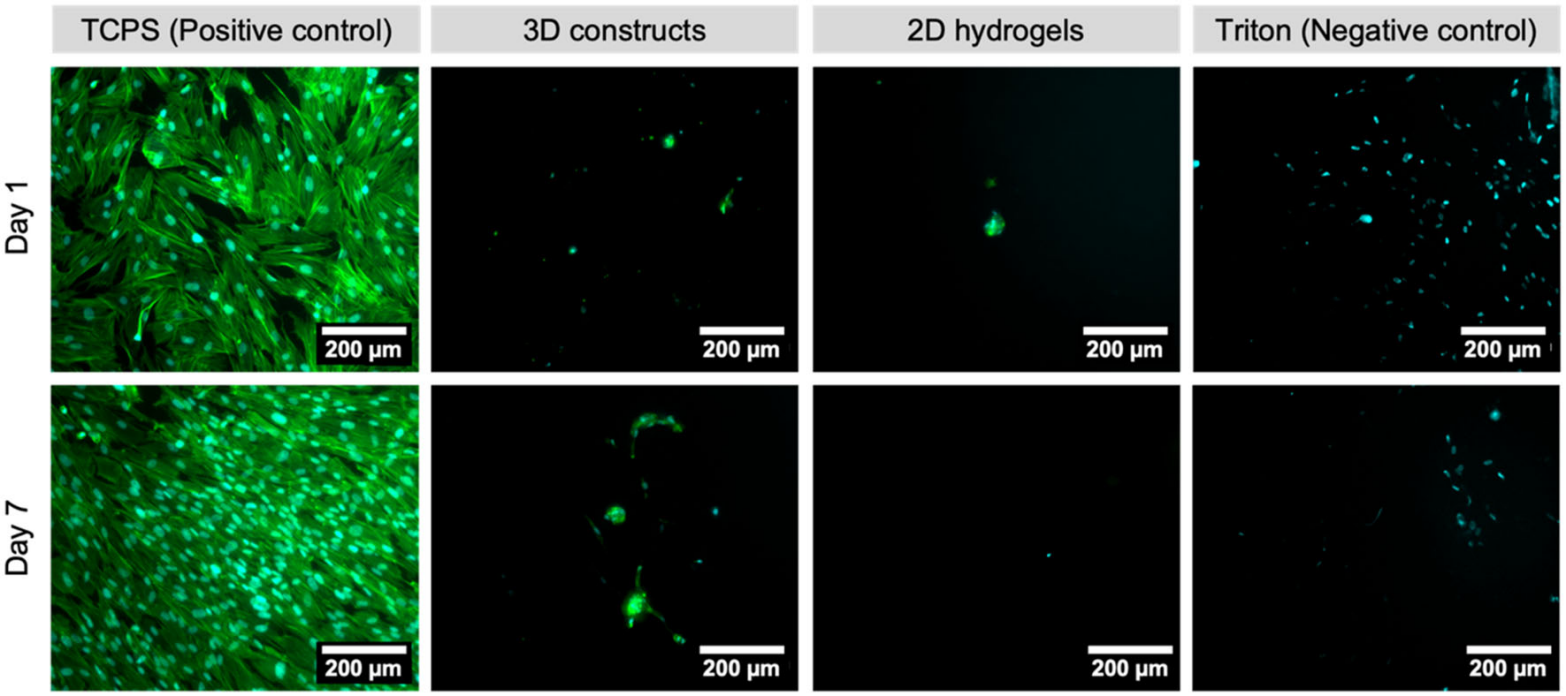
Fluorescence images of HFF‐1 fibroblasts seeded on PEG/GO 3D‐printed constructs and PEG/GO 2D hydrogels, at 1 (top row) and 7 (bottom row) days after seeding. Positive control: HFF‐1 seeded on tissue‐culture polystyrene; negative control: HFF‐1 seeded on TCPS and cultured with 0.2% w/v Triton X‐100 supplemented media. Actin cytoskeleton was stained with phalloidin (green), and cell nuclei were stained with DAPI (cyan). Scale bar: 200 μm.

To investigate if the CLADDING support bath remnants altered the surface properties of the 3D‐printed PEG/GO constructs, HFF‐1 fibroblasts were seeded on top of these samples and maintained for up to 1 and 7 days. Analyzing microscopic fluorescence images of phalloidin/DAPI staining (**Figure** **8**), it is possible to see that, on TCPS (positive control), fibroblasts proliferated and elongated, which is also confirmed by the metabolic activity measurements of the cells (Figure S7A, Supporting Information). On the other hand, very few cells adhered to 3D‐printed PEG/GO constructs, as can be seen in fluorescence images (Figure 8). 3D constructs appear to be as anti‐adhesive as 2D PEG/GO hydrogels produced by molding. Metabolic activity results (Figure S7B, Supporting Information) complement these observations. On day 1, the metabolic activity of HFF‐1 cells seeded on 3D‐printed PEG/GO constructs or 2D PEG/GO hydrogels was 6.8 ± 2.0% and 3.4 ± 2.5%, respectively (vs. cells seeded on TCPS); those values decreased to 3.4 ± 2.5% and 4.3 ± 3.3%, respectively, on day 7. Hence, the metabolic activity of HFF‐1 was similarly low for 2D hydrogels and 3D constructs, evidencing a similar antiadhesive behavior.

**Figure 9:**
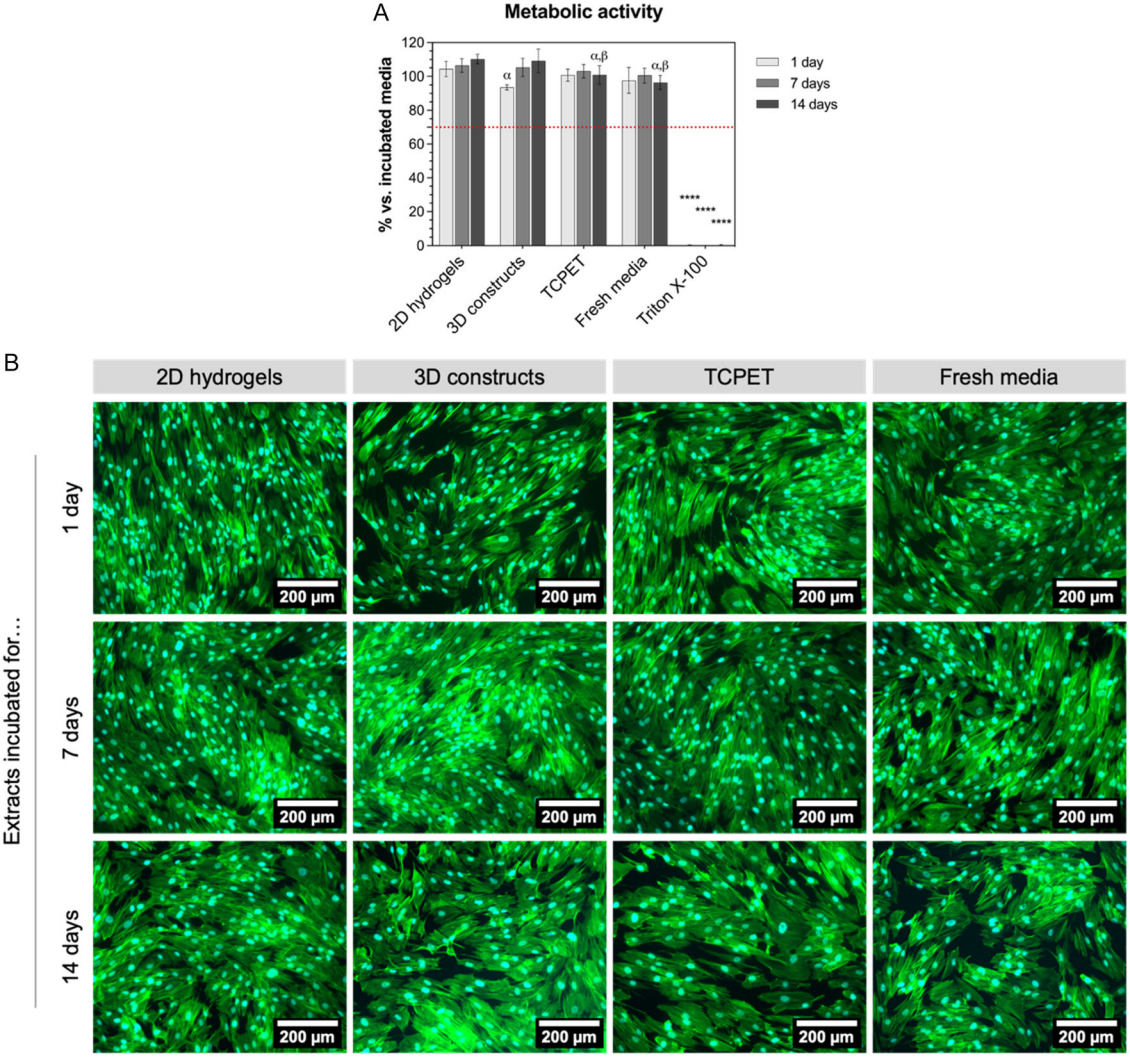
Cytocompatibility of extracts of biomaterials. A) Metabolic activity of HFF‐1 fibroblasts incubated with extracts of PEG/GO 2D hydrogels, 3D‐printed PEG/GO constructs, TCPET (reference material) incubated for 1, 7 or 14 days, fresh media (positive control) or 1% w/v Triton X‐100 (negative control), at 1 day after seeding; values presented as percentage normalized relative to HFF‐1 cultured with culture media incubated for the correspondent extracts’ period (which represents ≈100% of metabolic activity). Mean ± SD, *n* = 5 replicates, One‐Way ANOVA, ****p < 0.0001 versus all other conditions of respective timepoint, α: *p < *0.05 versus 2D hydrogels of respective timepoint, β: *p *< 0.05 versus 3D constructs of respective timepoint. B) Fluorescence images of HFF‐1 fibroblasts incubated with extracts of PEG/GO 2D hydrogels, 3D‐printed PEG/GO constructs, TCPET, or fresh media, for extracts incubated for 1 day (top row), 7 days (middle row), and 14 days (bottom row). Actin cytoskeleton was stained with phalloidin (green), and cell nuclei were stained with DAPI (cyan). Scale bar: 200 μm.

It is important to show that HFF‐1 fibroblasts indeed do not adhere due to the biomaterials’ surface properties—and not because these biomaterials are cytotoxic. To confirm this, the cytocompatibility of PEG/GO 3D‐printed constructs' extracts was assessed. 3D constructs and 2D hydrogels were incubated with cell culture media for 1, 7, and 14 days, at 37 °C and under agitation, to produce said extracts. HFF‐1 fibroblasts were then incubated with these media with extracts from each timepoint of incubation, and their metabolic activity and morphology were assessed. Results show that HFF‐1 fibroblasts incubated with extracts from either 3D constructs or 2D hydrogels had a metabolic activity close to 100% (vs. normal culture media) (**Figure** **9**A), for all timepoints, and also presented a regular stretched morphology (Figure 9B). These results show that extracts from 3D‐printed constructs were cytocompatible.

## 3 Discussion

Most works in literature exploring 3D printing of graphene‐containing biomaterials use low concentrations of graphene or GO (typically < 1% w/v).^[^ ^6^ ^]^ Furthermore, additional challenges come with the use of chemical crosslinkable polymers and the integration of crosslinking initiators in the traditional printing setup, which can be illustrated by the example of the PEG dimethacrylate/4% w/v GO ink crosslinked by a redox pair (APS and SMB) presented herein. This work overcomes these challenges by introducing a support bath that allows for shape retention of uncrosslinked PEG/GO filaments and a means to add the crosslinking redox initiators.

A screening of several support baths produced in‐house resulted in selecting the CLADDING support bath for printing PEG/GO ink. The Pluronic and Carbopol baths showed the formation of grooves that do not completely self‐heal, while agarose and FRESH v1 baths do not allow for the crosslinking of smooth filaments. These characteristics raise issues in the embedded 3D printing of multilayered geometries. On the contrary, the CLADDING support bath yielded the best results. Alongside FRESH, they have shown promising results regarding printing resolution of extremely low‐viscosity bioinks.^[^ ^28^, ^29^ ^]^ The CLADDING method provides a clear parametric fabrication window for the production of gelatin/gum arabic microparticles, using gelatin with defined gel strength and ion content, through a tightly regulated coacervation process.^[^ ^29^ ^]^ In this work, indeed, CLADDING showed the best self‐healing behavior and yielded smoother PEG/GO filaments, likely due to its spherical and smaller microparticles (compared to other tested baths). At such small sizes, the existence of microparticles in different sizes could even contribute to more stable ink deposition and greater printing resolution. Indeed, smaller particles potentially help fill up surrounding spaces in‐between larger microparticles, conferring the CLADDING support bath a micro–macro character, as previously hypothesized.^[^ ^29^ ^]^ In fact, this is only useful when the microparticle size is much lower than the intended printing resolution—here, filament width (≈500 μm) is around 16× larger than microparticle size (≈30 μm). Additionally, together these works show that the CLADDING support bath can be used to print inks with a viscosity in the 0.2–50 Pa s range (from 0.1 s^−1^ shear rate), at least.

Of notice, in this case, the choice of an appropriate support bath to print a desired ink should also take into account possible interactions between the ink and support bath's materials and how they could influence surface interface stability, printability, or even release of the constructs from the support bath. A recent and thorough paper by Lei and colleagues has delved into this and proposed guidelines for support bath selection by evaluating the printability and “shapeability” of various pairings of support baths and precrosslinked hydrogel inks.^[^ ^38^ ^]^ For example, anionic or neutrally‐charged photocrosslinkable inks—such as 700 Da PEG diacrylate with sodium hyaluronate—can be printed in Carbopol, agarose, or FRESH support baths (which are themselves anionic, neutral or cationic, respectively), preferably including the photoinitiator in both the ink and the bath (to minimize photoinitiator flux from the ink to the bath). This indicates that photopolymerization is faster than diffusion of the low‐viscosity polymeric ink. On the other hand, small monomeric inks crosslinked by free radical polymerization—such as 71.1 Da acrylamide/bisacrylamide—should be printed in an oil‐based support bath (to prevent monomer diffusion from the printed filament to the support bath), preferably including both free radical initiators in the ink. This indicates that diffusion of the low‐viscosity monomeric ink is faster than free radical polymerization. Our PEG/GO ink would fit in between these ink categories, as it is a chemically crosslinkable, 8 kDa polymeric ink, and potentially anionic (while PEG is an uncharged polymer, GO sheets have a negative zeta potential over a wide pH range) (Table S3, Supporting Information).^[^ ^40^ ^]^ Therefore, although also crosslinkable by a free radical initiation mechanism, polymer diffusion from the filament to support bath is not an issue in this case, and so aqueous‐based, cationic support baths can be used, such as CLADDING (Table S3, Supporting Information). As per the aforementioned considerations, this is an appropriate pairing of ink/bath.

Regarding printing resolution, this study is among the first to demonstrate the printing of inks with such a high concentration of GO (4% w/v) into features below 500 μm and, consequently, the first to show higher resolutions than previous related works.^[^ ^15^, ^23^, ^30^ ^]^

The nature of the interstitial phase of the CLADDING support bath has also been shown to have an impact on the geometrical and mechanical properties of the printing constructs—that is, even for the same ink and crosslinking mechanism. In this work, CLADDING support baths were prepared in MiliQ, SMB, APS, or CaCl_2_ solutions. Lower printing resolution in CLADDING prepared in MiliQ water highlights the importance of salt balance and concentration in the support bath interstitial phase, as previously shown.^[^ ^28^, ^29^ ^]^ Having one of the redox initiators in the support bath was expected to contribute to a faster and perhaps more complete crosslinking of the PEG/GO construct, but no such conclusions could be drawn. On the other hand, PEG/GO constructs produced in CLADDING in CaCl_2_ showed the best structural integrity and tensile properties. This may be explained by: 1) alterations in the hydrophobicity of the support bath, causing the PEG/GO ink to disperse less and conferring greater interlayer adhesion to the constructs; or 2) calcium ions working as an additional crosslinker between negatively‐charged GO sheets, resulting in stronger hydrogels.^[^ ^41^, ^42^, ^43^ ^]^ However, the embedded 3D printing process weakened the PEG/GO hydrogels’ overall mechanical properties. We have previously shown that 2D PEG/GO hydrogels produced by molding presented a 163 kPa Young's modulus, 218 kPa tensile strength and 126% elongation at break.^[^ ^32^ ^]^ In comparison, 3D‐printed PEG/GO constructs presented a 9.4 kPa Young's modulus, 68.2 kPa tensile strength and 158% elongation at break. Hence, the stiffness and strength of the 3D‐printed PEG/GO constructs were 94% and 69% lower, respectively, than those of 2D PEG/GO hydrogels. Although this direct comparison should be taken with reservations given the differences in sample geometry and test type, it illustrates how embedded 3D printing can affect the final mechanical properties of the material and construct, as already denoted in literature.^[^ ^14^ ^]^

To scout the possible biomedical applications for such a biomaterial, burst pressure and compliance are useful parameters. The PEG/GO constructs (particularly the strongest ones printed in CLADDING in CaCl_2_) could be applied as lymphatic grafts (physiological intrinsic contraction of lymphatic vessels occurs in the 12–70 mmHg range), for example.^[^ ^44^ ^]^ Results have shown that PEG/GO constructs are also anti‐adhesive toward endothelial cells (Figure S8, Supporting Information). Mechanical properties could be further improved by using PEG with lower molecular weights, or even by exploring other strategies for hydrogel mechanical reinforcement, such as interpenetrating polymer networks, double networks, or supramolecular‐interaction‐based hydrogels—all of which are compatible with the embedded 3D printing process.^[^ ^18^, ^38^, ^45^ ^]^ Yet, the advantages of this manufacturing method are multiple: starting from a PEG/GO ink that had suboptimal viscosity, slow crosslinking, and no shape retention, we were able to obtain stable constructs, with complex, micrometer‐wide, millimeter‐length 3D geometries, and mechanical properties in the kilo‐Pascal order of magnitude.

This interfacial interaction between the PEG/GO ink and CLADDING support bath also leads to support bath remnants on the surface of the constructs after retrieval and washing, as could be concluded from FTIR analysis. One must take into consideration the chemical nature and hydrophilicity of both ink and the support bath components. CLADDING is composed of microparticles stabilized by Pluronic, which adheres to the microparticles through its hydrophobic poly(propylene oxide) (PPO) domain and exposes its hydrophilic uncharged poly(ethylene oxide) (PEO) domains.^[^ ^46^ ^]^ PEO has a similar chemical composition to PEG, so it may interact with GO sheets present in the ink (via hydrogen bonds or electrostatic interactions). Additionally, because the CLADDING components are long polymeric chains with a high molecular weight, it is possible that these chains may be entangled/retained in the PEG/GO network at the constructs’ surface—especially considering that these PEG/GO hydrogels have a considerable swelling degree (≈800%). These interactions may explain the support bath remnants on the surface of the 3D‐printed PEG/GO constructs.

Of notice, 3D‐printing the PEG/GO ink inside a support bath leads to a less compact structure than producing the same construct by molding, for example. This could potentially lead to a greater release of GO nanosheets from the printed constructs and raise concerns about their cytocompatibility. However, the 3D‐printed PEG/GO constructs’ extracts were proven to be cytocompatible using HFF‐1 fibroblasts, suggesting good interlayer adhesion and stability of the constructs. Furthermore, these data are important to rule out the possibility of cytotoxicity in the direct contact assays—i.e., when HFF‐1 fibroblasts do not adhere to the surface of 3D‐printed PEG/GO constructs, that is indeed because the surface is anti‐adhesive. Indeed, despite the CLADDING support bath remnants identified at the surface of the 3D‐printed PEG/GO constructs, these were shown to remain antiadhesive (like the 2D hydrogels). This reiterates the potential for tunability of this biomaterial and manufacturing process, so that further modifications of the material to promote cell attachment, growth, controlled degradation, etc., can be made in a controlled manner.

## 4 Conclusion

3D printing of graphene‐containing biomaterials may prove challenging due to various aspects—from lack of shape retention to a limitation in graphene concentration for fast crosslinking. We showed how these issues may be overcome by processing chemically crosslinkable and highly concentrated GO‐containing inks using the embedded 3D printing technique. Support bath selection and printing setup design are key to achieving printability and high resolutions. PEG/4% w/v GO inks were printed in CLADDING support bath and crosslinked using redox initiators, hence creating, with precise control, stable, multilayered PEG/GO constructs with submillimeter‐range resolution and millimeter‐length, enhanced mechanical properties and anti‐adhesive surfaces. Our findings contribute to the innovative processing of graphene‐containing biomaterials, paving the way for their use in a variety of biomedical applications.

## 5 Experimental Section

5.1

5.1.1

##### Preparation of PEG/GO Ink

Graphene oxide was produced in‐house from oxidation and exfoliation of graphite (7–11 μm lateral size, per manufacturer's indication, American Elements, California, USA), by modified Hummer's method, as previously reported.^[^ ^47^ ^]^ PEG dimethacrylate powder (average molecular weight 8000 g mol^−1^, Ref. 25 428) was purchased from Polysciences (Hirschberg, Germany). To prepare PEG/GO suspensions, firstly, a 40 mg mL^−1^ (4% w/v) GO suspension in distilled water was prepared by centrifugation of a stock GO suspension (Avanti High‐Speed Centrifuge, JA‐25.50 rotor, 20 000 rpm, 15 min). The necessary mass of PEG dimethacrylate was added to the 4% w/v GO suspension to obtain a 15% w/v PEG/4% w/v GO suspension (herein PEG/GO ink). The mixture was vortexed vigorously for 1 min and sonicated in an ultrasonic bath (Bondelin Sonorex Digitec, 320 W) for 30 min before printing, to promote homogenization and GO flake's dispersion. Whenever stated, PEG/GO suspensions with redox initiators were also prepared, particularly by adding the necessary volume of 20% w/v stock solutions of APS (Sigma–Aldrich 248614) and/or SMB (Sigma–Aldrich 161519), to obtain PEG/GO suspensions with 0.025, 0.05, or 0.1% w/v of each initiator.

##### Rheological Characterization of PEG/GO Ink

Rheological characterization of PEG/GO ink was performed using a Kinexus Pro rheometer (NETZSCH), with a cone‐plate geometry (40 mm diameter, 0.5° cone angle), using 180 μL of sample. To evaluate the viscosity of these suspensions, flow sweeps were conducted between 10^−1^ and 10^3^ s^−1^ shear rate (10 points decade^−1^), with steady state sensing. Flow sweep curves were plotted as viscosity versus shear rate. To assess PEG/GO gelation with different concentrations of redox initiators, a viscometric assay at a constant shear rate of 1 s^−1^ was performed up to 60 min (1 point/sec). These assays were plotted as viscosity versus time. To assess the viscoelastic behavior, amplitude sweeps were conducted between 10^−2^ and 10^3^% amplitude strain (constant oscillation frequency 1 Hz, 10 points decade^−1^). Amplitude sweep curves were plotted as storage modulus (G′) and viscous modulus (G″) versus strain.

##### Production of Support Baths

A 23% w/v Pluronic F127 gel was produced as previously described.^[^ ^15^ ^]^ Pluronic F127 powder (Sigma–Aldrich P2443) was slowly added to distilled water at 4 °C, under magnetic stirring, to dissolve overnight. The cold solution was then poured into a Petri dish and brought to room temperature (RT) for 3–4 h, to ensure complete gelation, and used as the final support bath. A 0.5% w/v agarose fluid gel was produced as previously described.^[^ ^26^ ^]^ A 0.5% w/v agarose solution in distilled water was autoclaved. Immediately after that, it was put under magnetic stirring (700 rpm) while it cooled down to RT, for ≈3 h. The fluid gel was poured into a Petri dish and degassed under vacuum. A 1.2% w/v Carbopol 940P gel was produced as previously described.^[^ ^23^ ^]^ Carbopol 940P NF (Lubrizol, Ohio, USA) powder was dispersed in distilled water at RT, under magnetic stirring, for 3 h. Afterward, the Carbopol solution was neutralized with a 10 M NaOH (J.T. Baker 1310‐73‐2) solution, resulting in a gel that was loaded into a Petri dish and degassed under vacuum. FRESH v1 (which corresponds to a gelatin mechanical slurry) was produced as previously described.^[^ ^27^ ^]^ A 4.5% w/v gelatin type A in 150 mL of 11 mM CaCl_2_ (Avantor 1332‐01) solution was prepared and left to jellify at 4 °C overnight. After adding 350 mL of 11 mM CaCl_2_ solution, the gelatin gel was blended using a consumer‐grade blender to obtain a slurry. This mixture was centrifuged (Eppendorf 5810R centrifuge, 4200 rpm, 2 min) and resuspended in CaCl_2_ solution to obtain the final support bath, which was then transferred to a Petri dish and degassed under vacuum. The CLADDING support bath was produced based on a mixture of 20 g L^−1^ gelatin type B (Rousselot Biomedical, bovine bone LBB8, defined gel strength bloom 250), 5 g L^−1^ gum arabic (Sigma–Aldrich G9752) and 2.5 g L^−1^ Pluronic F127 in a 50% v/v ethanol/water solvent mixture, through a multistep coacervation process, as previously described.^[^ ^29^ ^]^ Briefly, 500 mL of MiliQ water was heated up to 45 °C under magnetic stirring (500 rpm), and 20 g gelatin and 5 g gum arabic were slowly added, leaving the mixture for 1 h to allow for total dissolution. Then, the solvent mixture was completed by adding 500 mL of absolute ethanol, followed by the addition of 2.5 g of Pluronic F127 (Sigma P2443), and further homogenization for 15 min. The pH was adjusted to 5.8 by the addition of 1 M HCl dropwise, triggering the coacervation of the gelatin and gum arabic molecules to form microparticles, noticed by a turbidity increase. The mixture was left at RT for 6 h, and then transferred to 4 °C for 12 h, allowing for precipitation of the coacervate. The supernatant was discarded, and the coacervate recovered and centrifuged (Eppendorf 5810R centrifuge, 800 rcf, 5 min) to remove solvent excessive remnants.

To produce the final support bath, this coacervate was dispersed in different solutions to obtain a colloid microparticle character, namely MiliQ water, or solutions of 0.1, 1% w/v APS, 1% w/v SMB, or 20 mM CaCl_2_ in MiliQ water—herein designated MiliQ‐, APS‐, SMB‐, or CLADDING in CaCl_2_, respectively, for simplicity. The coacervate was dispersed in each solution by vigorously shaking the tubes containing the mixtures, centrifuged (1000 rcf, 5 min), and the supernatant was discarded. This process was repeated three times, and the last centrifugation was performed at 2000 rcf, 5 min, to compact the microparticles and obtain the final support bath. The support bath was then transferred to a Petri dish or well plate with a spatula and degassed under vacuum.

##### Rheological Characterization of Support Baths

Rheological characterization of support baths was performed using a Kinexus Pro rheometer (NETZSCH) with a cone‐plate geometry (40 mm diameter, 0.5° cone angle), using 180 μL of sample. To assess the viscous behavior of these formulations, flow sweeps were conducted between 10^−1^ and 10^3^ s^−1^ shear rate (10 points decade^−1^), with steady state sensing. Flow sweep curves were plotted as viscosity versus shear rate. To analyze the self‐healing capacity of the support baths, a three‐step flow assay was performed. The sample was submitted to a 0.1 s^−1^ shear rate for 2 min, followed by 2 min of a 100 s^−1^ shear rate, and then down to a 0.1 s^−1^ shear rate for 2 min (1 point sec^−1^). These assays are plotted as viscosity versus time. To assess the viscoelastic behavior of the support baths, amplitude sweeps were conducted between 10^−2^ and 10^2^% amplitude strain (constant oscillation frequency 1 Hz, 10 points decade^−1^). Amplitude sweep curves were plotted as storage modulus (G′) and viscous modulus (G′) versus strain.

##### Microparticles Size Distribution of Support Baths

Microparticle size distribution was assessed by a dynamic light scattering (DLS)‐based technique, using a MasterSizer 3000 (Malvern Instruments Ltd., UK), with a wet Hydro LV dispersion unit and wet cell. Briefly, a beaker with ≈500 mL of distilled water was placed in the dispersion unit, under stirring. Support bath microparticle dispersions (prepared as aforementioned) were added using a Pasteur pipette to the beaker, until an obscuration of 5%–10% was achieved, and measurements were performed. Particle size was determined assuming a nonspherical particle type, refractive index of 1.5 for all samples, and presented as a volume distribution. Particle size distributions were not possible to obtain for Pluronic or Carbopol support baths for technical reasons, since obscuration target values were never achieved. From particle size distributions, particle diameter at percentile 10 (Dv10), 50 (Dv50, corresponding to median size), and 90 (Dv90) were calculated.

##### Microscopic Imaging of Support Baths

Dilutions of 1:80 of each support bath were prepared in MiliQ water and pipetted to a well plate immediately before imaging. Imaging was performed using an Axiovert 200M inverted widefield microscope (Zeiss, Jena, Germany) coupled to a monochromatic camera, with a 200× magnification and in phase‐contrast mode. Images were analyzed using ImageJ2 (version 2.14.0/1.54f).

##### 3D Printing of PEG/GO Ink

PEG/GO ink was 3D‐printed either without a support bath (i.e., directly on a Petri dish, “in air”), within solutions of dissolved initiators (1% w/v APS or 1% w/v SMB), or within support baths. Before printing, PEG/GO ink was loaded into a 3‐mL syringe and degassed by vacuum. Printing was performed using a Nordson Robot Pro Automated Dispensing System (Nordson EFD, Ohio, USA) through pneumatic extrusion using high‐precision pressure controllers. The geometries and printing paths were directly designed in the DispenseMotion software, in a DXF file format. Single filaments of PEG/GO ink of 2 cm were printed using blunt‐end 25G printing needles (Cellink NZ5250505001, 0.25 mm inner diameter, 0.52 mm outer diameter). Cylindrical constructs with 4 mm inner diameter and variable length were printed by deposition of stacked concentric ring layers of PEG/GO ink, using blunt‐end 27G printing needles (Cellink NZ5271005001, 0.2 mm inner diameter, 0.42 mm outer diameter), which ultimately determined filament width and the cylinders’ wall thickness. 3D printing parameters to be optimized included extrusion pressure (varied in 0.8–1.2 bar), printing speed (varied in 1–6 mm sec^−1^), interlayer spacing (varied in 0.1–0.2 mm), and number of layers (varied in 3–30 layers). All printing optimization was performed in CLADDING in APS support baths. After optimization, for the remaining work, PEG/GO constructs were printed using 1 bar extrusion pressure, 6 mm s^−1^, printing speed, 0.15 mm interlayer spacing, and 30 layers. After extrusion, 5 μL of highly concentrated redox initiator solutions (20% w/v APS and 20% w/v SMB) were pipetted directly on top of the extruded constructs, and these were left overnight to ensure initiators diffused through the support bath, initiating crosslinking and gelation was complete.

##### Releasing and Washing of PEG/GO Constructs

After crosslinking was completed, PEG/GO constructs were carefully removed from the support bath with a spatula, immersed in distilled water at 37 °C, and washed under orbital shaking (150 rpm), overnight. This temperature allows the gelatin to melt and disintegrate the support bath that remains on the surface of the constructs.

##### Imaging and Geometrical Characterization of PEG/GO Constructs

After washing, PEG/GO constructs were imaged using a stereomicroscope (Olympus), with a 12.5× magnification and over a darkfield background (top and side views). Images were analyzed using ImageJ2 (version 2.14.0/1.54f) and used to measure the dimensions of the printed constructs—namely inner diameter and wall thickness (or filament width) from top view images, and length from lateral view images (when applicable).

##### Surface Characterization of PEG/GO Constructs

To assess if washing was successful, the surface chemical composition of PEG/GO constructs was analyzed by FTIR, in ATR mode. 3D‐printed PEG/GO constructs before and after washing, CLADDING support bath, and 2D PEG/GO hydrogels were dried in a vacuum oven (25 °C, overnight). Their FTIR‐ATR spectra were acquired using a Frontier FTIR equipment (Perkin Elmer), in the 400–2000 cm^−1^ wavenumber range (4 cm^−1^ resolution, 32 scans). FTIR spectra were plotted as transmittance versus wavenumber.

##### Mechanical Characterization of PEG/GO Constructs

PEG/GO cylindrical constructs were mechanically characterized by uniaxial ring‐hoop tensile tests until rupture, using a TA.XTplus Texture Analyzer (Stable Micro Systems, Surrey, UK), with a load cell of 5 kg. PEG/GO cylindrical constructs (with ≈3 mm inner diameter, 0.5 mm wall thickness, and 4.5 mm length) were loaded around two steel 0.6 mm‐thick pins, and the tensile tests were conducted at a displacement rate of 10 mm min^−1^ until disruption of the constructs. Stress–strain curves were acquired for all samples, and tensile properties were calculated, namely: Young's modulus (YM), as the slope of the linear regression in the 0–0.2 mm mm^−1^ strain range; ultimate tensile strength (UTS), as the maximum stress registered immediately before the break; and elongation at break (EB) as the correspondent strain immediately before break.

##### Antiadhesiveness of PEG/GO Constructs Toward Human Fibroblasts

To observe how the printing process affected the PEG/GO surface properties, fibroblast adhesion was studied. PEG/GO constructs 3D‐printed in CLADDING in CaCl_2_, and 2D PEG/GO films prepared by molding, as previously described, were used.^[^ ^32^ ^]^ Firstly, 5‐mm disc samples were cut from either 3D‐printed PEG/GO constructs or 2D PEG/GO films, sterilized with 70% ethanol for 30 min, and rinsed with sterile phosphate‐buffered saline (PBS).

Human foreskin fibroblasts (HFF‐1, cell line ATCC SCRC1041) were thawed and maintained in DMEM (Gibco 21885‐025, GlutaMAX, low glucose, pyruvate) supplemented with 10 vol% fetal bovine serum (Gibco 10270‐106) and 1 vol% penicillin/streptavidin (Pan Biotech P06‐07100). Upon reaching ≈80% confluency, cells were trypsinized, centrifuged (1200 rpm, 5 min), collected, and counted using a Neubauer chamber for cell seeding.

Sterile 5‐mm discs of each material (3D‐printed PEG/GO constructs or 2D PEG/GO films) were placed in 96‐well suspension plates, and HFF‐1 (1 × 10^5^ cell mL^−1^, 100 μL) were seeded on these discs. Cells were allowed to adhere and maintained in culture for up to 1 and 7 days (37 °C, 5% CO_2_). At these time points, the metabolic activity of adherent cells was evaluated by resazurin assay. To do so, samples were transferred to new 96‐well suspension plates and incubated with supplemented DMEM with 20 vol% resazurin (Sigma R7017‐1G) for 4 h. Fluorescence (*λ*_excitation_: 530 nm, *λ*_emission_: 590 nm) was measured using Synergy Mx microplate reader (BioTek Instruments, CA, USA). Additionally, HFF‐1 adhesion and morphology were assessed by phalloidin/DAPI staining. Cells were fixed with 4% w/v paraformaldehyde for 20 min and rinsed 3× with PBS. Permeabilization was performed with 0.1 vol% Triton X‐100 (VWR 0694) for 5 min, after which they were rinsed with PBS. Cells were then stained with 10 μg mL^−1^ phalloidin (Alexa Fluor 488, Thermo Fisher Scientific A12379) for 30 min, RT. Nuclei were counterstained with 3 μg mL^−1^ DAPI for 15 min, RT, followed by PBS rinsing. Imaging was performed using an Axiovert 200M inverted widefield fluorescence microscope (Zeiss, Jena, Germany) coupled to a monochromatic camera, with a 100× magnification. Images were analyzed using ImageJ2 (version 2.14.0/1.54f).

##### Cytocompatibility of PEG/GO 3D Constructs Extracts

PEG/GO constructs 3D‐printed in CLADDING in CaCl2 and 2D PEG/GO hydrogels (Ø = 5 mm) were sterilized with 70% ethanol and rinsed with sterile PBS. Extracts were prepared according to International Standard ISO 10993‐12, reflecting a 0.5–1.0 mm thickness and using a surface area‐to‐volume extraction ratio of 3 cm^2^ mL^−1^.^[^ ^48^ ^]^ Briefly, eight 5 mm‐diameter discs of each sample were incubated with supplemented media. Incubation was carried out for 1, 7 and 14 days at 37 °C, in an orbital shaker at 100 rpm. Tissue‐culture polyethylene terephthalate (TCPET) was used as a reference material with no cytotoxicity. Culture medium without any material was incubated under the same conditions (herein denoted as “24 h medium”). The extracts assay was performed according to ISO 10993‐5.^[^ ^49^ ^]^ HFF‐1 fibroblasts (1 × 10^5^ cells mL^−1^, 100 μL) were seeded on TCPS wells of 96‐well plates and incubated for 24 h (37 °C, 5% CO2). After that, the culture medium was replaced by 100 μL of materials’ extracts. Control conditions included HFF‐1 cultured with incubated medium, fresh medium, and medium with 1 vol% Triton X‐100. Metabolic activity was assessed at 24 h by a resazurin assay, and HFF‐1 morphology was assessed by DAPI/phalloidin staining, as mentioned in the previous section.

##### Statistical Analysis

Graphs were prepared using GraphPad (version 9.5.1). Datasets were tested for Gaussian distribution using the Shapiro–Wilk normality test (*α *= 0.05). Unless stated otherwise, statistical differences between groups were calculated based on ordinary one‐way ANOVA, followed by Tukey's multiple comparison tests. A *p*‐value lower than 0.05 was considered statistically significant. Data are shown as mean ± standard deviation (SD) or represented using standard deviation bars in the graphs.

## Supporting Information

Supporting Information is available from the Wiley Online Library or from the author.

## Conflict of Interest

The authors declare no conflict of interest.

## Supporting information

Supplementary Material

## Data Availability

The data that support the findings of this study are available from the corresponding author upon reasonable request.
