# Supplementary material for: Embedded 3D Printing of Graphene Oxide‐Containing, Chemically Crosslinkable Poly(Ethylene Glycol) Inks
Source: Small Sci. 2025 Nov 8;5(12):e202500278. doi: 10.1002/smsc.202500278 (PMC12697804; doi:10.1002/smsc.202500278)
Supplement: Supplementary file 1 — Supplementary Material [file SMSC-5-e202500278-s001.pdf]

Supporting Information

Embedded 3D printing of graphene oxide-containing, chemically crosslinkable poly(ethylene glycol) ink

Helena P. Ferreira, Monize C. Decarli, Duarte Moura, Rúben F. Pereira, Andreia T. Pereira, Lorenzo Moroni#, Inês C. Gonçalves *#*

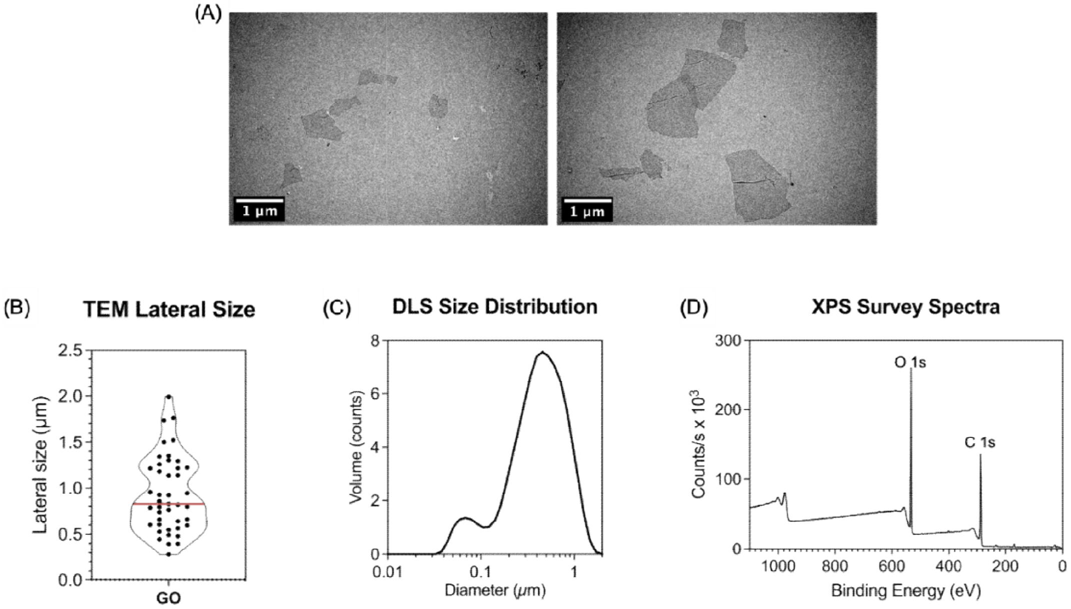

(E)

Summary of GO characterization	
TEM lateral size (mean ± SD)	0.934 ± 0.408 µm
DLS size distribution (peaks and PdI)	Avg. Peak 1: 0.0681 µm<br>Avg. Peak 2: 0.531 µm<br>PdI: 0.41
Zeta potential (mean ± SD)	- 47.3 ± 0.51 mV
Conductivity (mean ± SD)	0.0224 ± 0.0044 mS/cm
Oxidation degree (XPS)	28.5%

Figure S 1. GO characterization. (A) TEM images of GO particles; scale bar: 1 µm. (B) Lateral size of GO sheets, as per TEM images measurements (mean value, n=2 replicates). (C) DLS size distribution of GO suspension (n=3 replicates). (D) XPS survey spectra, with identification of characteristic peaks of carbon (284.8 eV) and oxygen (532.8 eV). (E) Summary of GO characterization from different techniques, namely TEM, DLS, zeta-potential measurements and XPS.

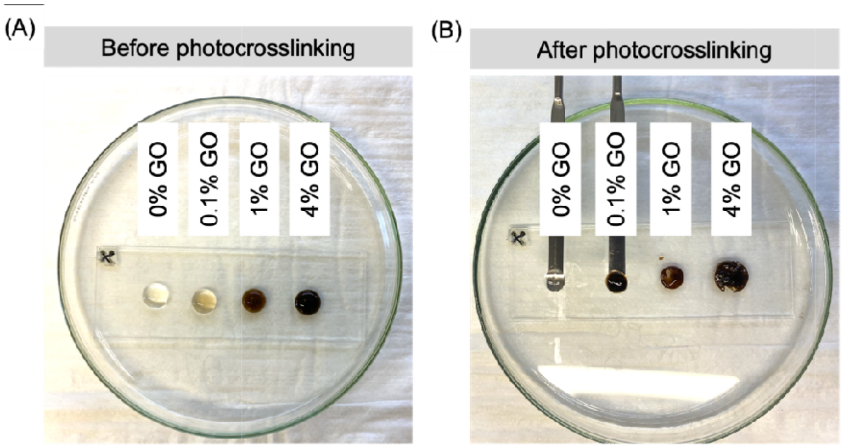

Figure S 2. Photocrosslinking of PEG/GO formulations. Images of PEG/GO formulations with 15% w/v PEG and 0, 0.1, 1 or 4 % w/v GO (and 0.5 % w/v Irgacure 2959 photoinitiator) either (A) before or (B) after photocrosslinking with UV light (365 nm, 30 mW/cm^2) for 30 min. It is possible to see that 0% and 0.1% GO formulations were photocrosslinked and formed a solid-like hydrogel (presented in the spatula), while formulations with > 1% GO did not photocrosslink.

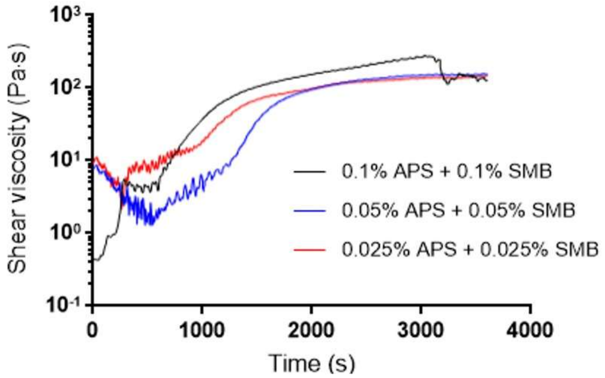

Figure S 3. Viscosity vs. time assay for PEG/GO ink with various concentrations of APS and SMB (0.1, 0.05, 0.025 % w/v).

Table S 1. Microparticle size distribution of different support baths. Dv10, Dv50 and Dv90 correspond to sizes where 10%, 50% or 90% of particles, respectively, fall below such value (Dv50 is the median of distribution).

**Support baths**	**Dv10 (µm)**	**Dv50 (µm)**	**Dv90 (µm)**
Agarose	34.9 ± 0.1	53.6 ± 0.1	83.5 ± 0.5
FRESH v1	78.4 ± 3.0	539 ± 26.9	1580 ± 102
CLADDING	13 ± 0.2	33.1 ± 0.8	90.3 ± 4.0

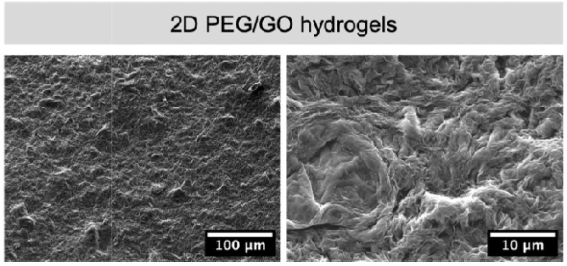

Figure S 4. SEM images of 2D PEG/GO hydrogels produced by molding.

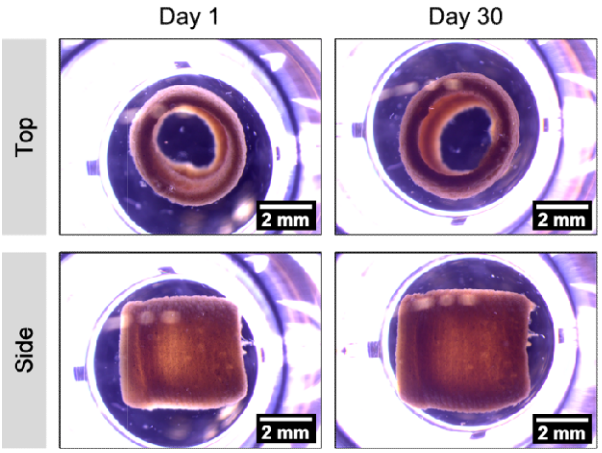

Figure S 5. Stereomicroscope images (top and side view) of PEG/GO constructs printed in CLADDING support baths, 1 day and 30 days after printing. Scale bar: 2 mm.

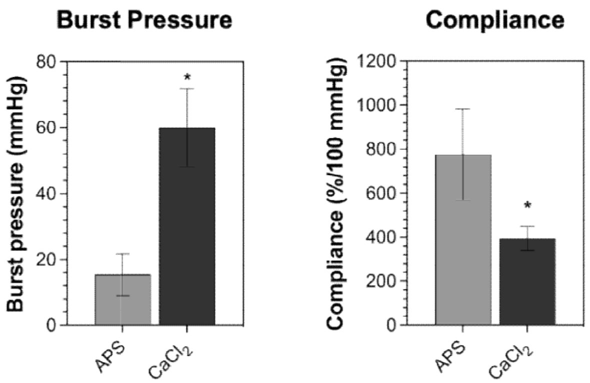

Figure S 6. Burst pressure and compliance of PEG/GO constructs printed in CLADDING baths in APS or $CaCl_2$; Mean±SD, n=8 replicates for each condition, parametric t-test, * $p<0.05$.

Table S 2. Dimensions of CAD model and PEG/GO constructs 3D-printed in CLADDDING baths in APS or $CaCl_2$. Values presented as mean ± SD, in mm. * Wall thickness is projected to have around 0.5 mm, but it is not included in the model, but rather controlled by the printing needle dimensions and printing parameters such as extrusion pressure and printing speed.

**Dimensions**	**CAD model (projected)**	Constructs from CLADDING in…	
		**APS**	**$CaCl_2$**
Wall thickness	~0.5 mm*	0.56±0.07	0.47±0.06
Inner diameter	4 mm	2.83±0.29	3.02±0.16
Length	4.5 mm	4.45±0.09	4.24±0.06

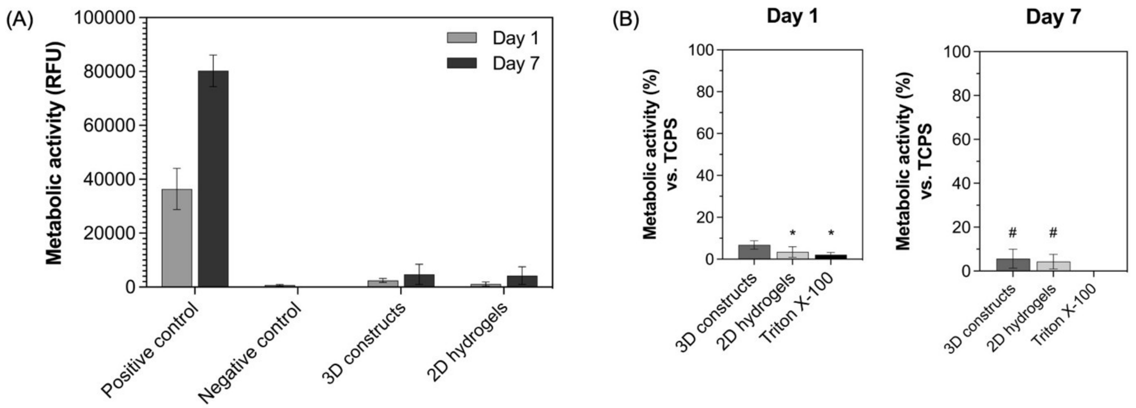

Figure S 7. (A) Metabolic activity (presented as random fluorescence units) of HFF-1 fibroblasts seeded on 3D-printed PEG/GO constructs, PEG/GO 2D films, positive control (HFF-1 seeded on TCPS) and negative control (HFF-1 seeded on TCPS and cultured with 0.2 % w/v Triton X-100 supplemented media), at 1 and 7 days after seeding. (B) Metabolic activity of HFF-1 fibroblasts seeded in each condition, presented as percentage normalized in relation to HFF-1 seeded on TCPS (positive control, which represents ~100% of metabolic activity). Mean±SD, n= 9 replicates, One-Way ANOVA, Kruskal-Wallis test, * $p< 0.05$ vs. 3D constructs, # $p< 0.05$ vs. Triton X-100.

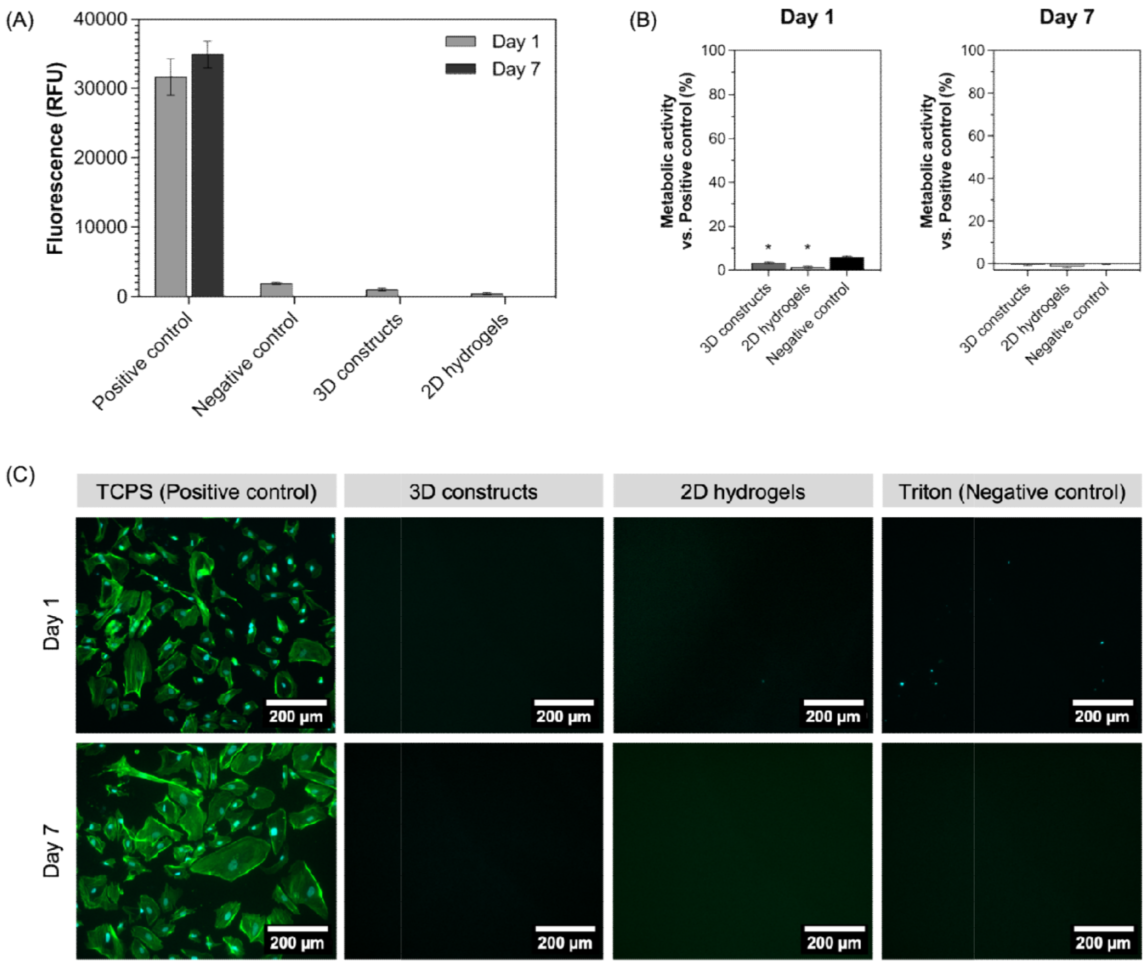

Figure S 8. Anti-adhesiveness towards human umbilical vein endothelial cells (HUVEC). (A) Metabolic activity (presented as random fluorescence units) of HUVEC seeded on 3D-printed PEG/GO constructs, PEG/GO 2D films, positive control (TCPS) and negative control (incubated with 0.2 % w/v Triton X-100 supplemented media), at 1 and 7 days after seeding. (B) Metabolic activity of HUVEC seeded in each condition, presented as percentage normalized in relation to HUVEC seeded on TCPS (positive control, which represents ~100% of metabolic activity). Mean+SD, n= 5 replicates, One-Way ANOVA, Kruskal-Wallis test, * $p< 0.05$ vs. negative control. (C) Fluorescence images of HUVEC stained with phalloidin for actin cytoskeleton (green) and cell nuclei were stained with DAPI (cyan); scale bar: 200 µm.

Table S 3. Comparison of characteristic of PEG/GO ink and CLADDING support bath.

	PEG/GO ink	CLADDING support bath
Composition and MW/size	15 % w/v PEG dimethacrylate (8 kDa)<br>4 % w/v GO (0.934 µm)	Microparticles (33.1 µm) of 4:1:0.5 of:<br>○ Gelatin type B (157.05 kDa)<br>○ Gum arabic (~250 kDa)<br>○ Pluronic F-127 (~12.5 kDa)
Putative charge	Anionic	Cationic
Viscosity (at 1 s^{-1} shear rate)	6.85 Pa·s	50.8 Pa·s
